# An integrated ChIP-seq analysis platform with customizable workflows

**DOI:** 10.1186/1471-2105-12-277

**Published:** 2011-07-07

**Authors:** Eugenia G Giannopoulou, Olivier Elemento

**Affiliations:** 1 HRH Prince Alwaleed Bin Talal Bin Abdulaziz Alsaud Institute for Computational Biomedicine, Weill Cornell Medical College, 1305 York Avenue, New York, NY, 10021, USA; 2 Department of Physiology and Biophysics, Weill Cornell Medical College, 1300 York Avenue, New York, NY, 10021, USA

## Abstract

**Background:**

Chromatin immunoprecipitation followed by next generation sequencing (ChIP-seq), enables unbiased and genome-wide mapping of protein-DNA interactions and epigenetic marks. The first step in ChIP-seq data analysis involves the identification of peaks (i.e., genomic locations with high density of mapped sequence reads). The next step consists of interpreting the biological meaning of the peaks through their association with known genes, pathways, regulatory elements, and integration with other experiments. Although several programs have been published for the analysis of ChIP-seq data, they often focus on the peak detection step and are usually not well suited for thorough, integrative analysis of the detected peaks.

**Results:**

To address the peak interpretation challenge, we have developed ChIPseeqer, an integrative, comprehensive, fast and user-friendly computational framework for in-depth analysis of ChIP-seq datasets. The novelty of our approach is the capability to combine several computational tools in order to create easily customized workflows that can be adapted to the user's needs and objectives. In this paper, we describe the main components of the ChIPseeqer framework, and also demonstrate the utility and diversity of the analyses offered, by analyzing a published ChIP-seq dataset.

**Conclusions:**

ChIPseeqer facilitates ChIP-seq data analysis by offering a flexible and powerful set of computational tools that can be used in combination with one another. The framework is freely available as a user-friendly GUI application, but all programs are also executable from the command line, thus providing flexibility and automatability for advanced users.

## Background

The use of chromatin immunoprecipitation in combination with high-throughput sequencing (ChIP-seq) has enabled the study of genome-wide mapping of protein-DNA interaction and epigenetic marks. By sequencing millions of immunoprecipitated DNA fragments in a single experiment, ChIP-seq outperforms the array-based ChIP-chip (Chromatin Immunoprecipitation followed by DNA microarray hybridization) technology in terms of quality, specificity, and coverage [1-3], and has the potential to greatly improve our understanding of the mechanisms underlying transcriptional regulation [4-8]. Many peak detection methodologies and software tools have been developed for the analysis of ChIP-seq data since the introduction of the technology [2,3,9,10]. Although peak detection is important for the analysis of ChIP-seq data, it is only the first step. Additional computational tools are needed to help interpret the genome-wide transcription factor binding and histone mark enrichment patterns revealed by peak detection procedures. We have developed ChIPseeqer, a comprehensive computational framework that enables broad, but also in-depth, analysis of ChIP-seq data. The framework includes: (1) gene-level annotation of peaks, (2) pathways enrichment analysis, (3) regulatory element analysis, using either a de novo approach, known or user-defined motifs, (4) nongenic peak annotation (repeats, CpG islands, duplications, published ChIP-seq datasets), (5) conservation analysis, (6) clustering analysis, (7) visualization tools, (8) integration and comparison across different ChIP-seq experiments. These components share a common architecture: they take as input a set of ChIP-seq peaks, perform the defined analysis, and output one or more sets of peaks that can be used by any of the other tools provided. Thus, our framework was designed to offer the flexibility required to perform complex analyses by defining custom workflows. In principle, ChIPseeqer can use peaks generated by any peak-calling program as initial input, assuming these peaks are in a simple and standard format (i.e., chromosome, start position, end position). For convenience, ChIPseeqer also includes its own fast and accurate peak finding algorithm, previously evaluated and compared with other algorithms in Qin et al. [11]. Details and additional validation of our peak detection algorithm are provided in Additional file 1. By analyzing a published ChIP-seq dataset, we show how the modular nature of the framework can help the users create easily computational pipelines and address sophisticated biological questions.

### Comparison with related work

Although numerous approaches already exist for the analysis of ChIP-seq data, many of them focus on the peak detection process and provide few or no tools for the interpretation of ChIP-seq peaks [5-8,12-19]. Several frameworks for interpreting ChIPseq datasets have nonetheless been developed, all of which have strengths and limitations. For example, the Cistrome project (unpublished, [20]) integrates several analysis tools, such as gene annotation, motif and pathways analysis, and peak conservation. However, users must upload their ChIP-seq data to the Cistrome server, which will be increasingly time-consuming and less practical as the size of ChIP-seq datasets increases. Moreover, feeding the results of an analysis directly into another tool within the same framework can be difficult for some users because each tool supports different input formats. Galaxy [21-25], which is used as Cistrome's backend, is a powerful framework but possibly too general for the analysis of ChIP-seq data. In particular, Galaxy does not allow control over several important parameters in ChIP-seq data analysis, such as the maximum or minimum distance between the peak and its closest gene in the peak-gene association task [26]. CisGenome [27] also supports tools for the integrated analysis of ChIP-seq data, such as annotation of peaks with their neighbor genes, conservation analysis, and motifs discovery. However, CisGenome does not allow the correlation of peaks and their target genes with Gene Ontology (GO) terms and pathways that could shed light on the biological processes and pathways controlled by the transcription factors (TFs) or histone modifications assayed by ChIP-seq. The GPAT program [26] provides systematic annotation of genomic positions in general. It uses gene annotation from different public databases, such as RefSeq and Ensembl, and provides access to the expression status of the corresponding genes from existing transcriptomic databases, or user-generated expression datasets. The limitation of GPAT is the lack of other tools for the analysis of ChIP-seq data, apart from genomic annotation. Thus, GPAT users cannot perform motif discovery, pathways enrichment, and other analyses that are useful in the ChIP-seq context. EpiChIP [28] offers gene-based enrichment analysis of ChIP-seq datasets. In particular, EpiChIP looks for enrichment of the ChIP-seq reads over the control sample in specific regions of the genes, such as the 5'- or 3'- end, exons or introns. This approach has the advantage of identifying directly the genes that are enriched in the TF or the histone modification of the reference dataset. However, the program lacks in providing further annotation of the enriched regions. The seqMINER platform [29] aims at integrating and comparing different ChIP-seq datasets in terms of read density. The algorithm first estimates for a set of genomic regions (i.e., reference dataset) the read density of multiple ChIP-seq datasets. Clustering and visualization methods are then provided to show groups of regions with similar binding features. Although this approach is useful to integrate ChIP-seq datasets, it focuses on the comparison of read density profiles and does not integrate other sources of information, such as the motifs and pathways enrichment or the level of conservation. HOMER [30] provides a suite of programs originally developed for motif discovery, and later for ChIP-seq peak detection. Although it includes tools for gene annotation, clustering, and visualization of the peaks (e.g., histograms, heatmaps), it does not support conservation analysis and can only run from the command-line. BEDTools [31] is a UNIX-based collection of utilities that allow common operations on genomic features in general (e.g., find overlaps between two files with genomic intervals, extract FASTA sequences from genomic intervals). Although the BEDTools are designed to provide fast solutions to basic operations on large data volumes produced by DNA sequencing, they do not offer computational tools for the functional interpretation of ChIP-seq peaks (e.g., motifs and pathways analysis). Their command-line nature also demands extra effort and computer skills from users. CEAS [32] is a stand-alone extension of a web application previously developed for ChIP-chip data [33], but is also offered through the Cistrome framework. The tool provides basic annotation tools for ChIP-seq data, such as the estimation of peaks distribution across the genome, identification of genes associated with peaks by proximity, and more. However, one drawback of CEAS, when using it through the Cistrome application, is that it produces graphical representation of the results and does not output lists of peaks that belong to specific genomic categories (e.g., promoters, introns). PeakAnalyzer [34] can subdivide ChIP-seq peaks that have multiple sites of enrichment into smaller peaks; this procedure may facilitate more detailed analysis of individual subpeaks. It also offers annotation functions, and can locate the nearest downstream genes and transcription start sites for each peak. It can also determine overlapping peaks between different datasets. Although PeakAnalyzer allows the user to perform gene annotation, motifs analysis, annotation with functional elements, and comparisons across datasets, it does not support the functional interpretation of the ChIPseq results through their association with pathways. On the other hand, GREAT [35] is a web application that supports the analysis of functional significance of ChIP-seq peaks using 20 different information sources (e.g., Gene Ontology, PANTHER pathway, Pathway Commons, InterPro). Importantly, the tool integrates not only proximal but also distal binding events to obtain a gene-based p-value for enrichment [35]. However, GREAT does not offer an automated way to retrieve lists of genes and peaks associated with a specific pathway, Gene Ontology term, or motif. This feature (provided in our framework) would enable users to perform further and more targeted analysis on subsets of the initial peaks, which were found to be functionally significant. In contrast, ChIPseeqer has many advantages compared to these programs. First, it offers a variety of tools that cover not only basic gene annotation, but also a wide range of computational analyses, including motif analysis, pathways enrichment, estimation of conservation, read density analysis and more. Second, ChIPseeqer allows the comparison of multiple datasets based not only on read density profiles, but also on peak binding overlap, and integration with other ChIP-seq datasets. Third, the framework provides a straightforward and effortless connection between the tools; no data format transformation is needed to combine the tools and perform a comprehensive and sophisticated data analysis. Fourth, the framework allows the users to control all parameters of the analysis, such as the minimum distance away from transcripts, the upstream distance from transcription start site (TSS), the database annotation and more. In addition, ChIPseeqer runs locally on the user's computer enabling the analysis of very large datasets. Finally, it provides a user-friendly graphical interface that can be used effortlessly even by non-expert users.

## Implementation

### Software distribution and availability

ChIPseeqer is available as a set of standalone command line tools. For advanced users, command line tools provide great flexibility and automatability. For less advanced users, we have made these tools available via a graphical user interface (GUI), developed using the multi-platform QT framework [36]. The bundle (i.e., command line tools and GUI) has been tested on Linux and Mac OS X. Detailed installation instructions and documentation for all tools included in the framework are also available online [37]. Our implementation is available as free software, released under the GNU General Public License (GPL) v3 [38].

### Comparison of genomic intervals

Many computations performed in ChIPseeqer involve assessing overlaps between hundreds or thousands of genomic regions (i.e., peaks, transcripts/genes, gene parts), and therefore, efficient algorithms are needed to quickly determine and characterize these overlaps. In ChIPseeqer, fast comparison on genomic intervals is performed using interval trees [39,40], ordered tree structures that store and index intervals with fast querying and processing times, and ensure efficient searching of all indexed intervals that overlap with any given interval or point. An interval tree is an augmented binary search tree: each node contains an interval and also stores the maximum endpoint of the subtree rooted at the particular node. Apart from the insert and delete operations that characterize the binary search trees, interval trees also support a query operation that allows searching the tree for overlaps with a given interval. The first step of the algorithm sets the root of the tree as current node. The second step checks if the given interval overlaps with the current node; if not, it compares the low endpoint of the given interval with the maximum value stored at the left child of the current node. If the low endpoint of the interval is lower than the maximum value stored, then the current node is set to the left child; otherwise it is set to the right child. Then the algorithm goes back to the first step and repeats the same procedure for the new current node until it finds an overlap of the given interval with the interval stored in the current node, or until the whole tree is explored. Of note, we are using a modified implementation of the original algorithm [40], so as not to stop at the first overlapping interval but find all intervals that overlap with the given one. Moreover, we use a randomization procedure that takes into account the natural clumping of features [41] to assess the statistical significance of the observed number of overlapping peaks between two peak files. This procedure consists of generating many "random" lists of peaks maintaining peak sizes and number of peaks as well as the chromosomal and genomic distribution of the peaks in the first peak file. The latter means that each random list of peaks maintains the same fraction of peaks in promoter, exonic, intronic, downstream, and intergenic regions as the original peak file. Then, for each random peak list, the number of overlapping peaks with the second peak file (kept unchanged) is calculated. A p-value is determined by counting the number of times the random overlap is equal to or greater than the originally observed number of overlapping peaks. Additionally, the z-score is estimated, representing the distance (in number of standard deviations) betweem the observed number of overlapping peaks and the average number of overlapping peaks expected by chance.

### Supported formats, annotations, and species

One of the advantages of the framework is the support of different formats that are well-established in deep sequencing experiments, such as *SAM*, *BAM*, *eland*, *extended eland*, *bed *and *export*. ChIPseeqer also provides gene-based annotation from multiple sources and databases, such as *RefSeq*, *Ensembl*, *UCSC Genes*, and *AceView*. Finally, four species are currently supported, namely *Homo sapiens*, *Mus musculus*, *Drosophila melanogaster*, and *Saccharomyces cerevisiae*. Support for additional species can be added to the framework as described in Additional file 1 and in our online documentation.

## Results

### ChIPseeqer user-defined workflows

**Figure 1:**
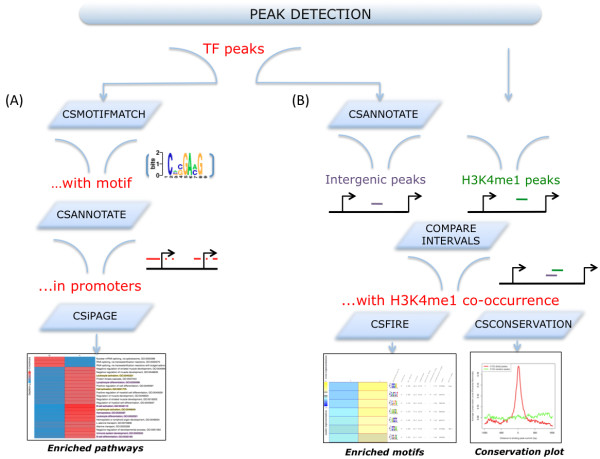
**Workflow use cases**. Examples of workflows that can be easily generated using tools from the ChIPseeqer framework are shown. The starting point is always the result of peak detection: a set of enriched regions/peaks. (A) The aim of the workflow is to analyze a subset of the peaks that have a specific motif. From all the peaks that have the motif, we look for those that bind in the promoters of known genes. Pathways analysis is then performed on these genes in order to reveal enriched pathways associated with this particular subset of peaks. (B) This workflow allows locating and characterizing distal regulatory elements (i.e., intergenic peaks) that overlap with enhancer marks (e.g., H3K4me1 binding), in terms of motifs and conservation. Different workflows can be created using any combination of the ChIPseeqer tools.

ChIPseeqer is a comprehensive and fully integrated framework offering a dry-lab workbench for the processing and analysis of ChIP-seq data. Table 1 summarizes the basic tools in the framework along with a short description of their functionality and their availability in the ChIPseeqer interface. A detailed description of these tools is provided in the *ChIPseeqer modules *section. The framework includes a peak detection program as well as tools for performing quality control of the raw reads (see Additional file 1). However, the most interesting aspect of ChIPseeqer is the variety of independent analysis modules, all of which have the same structure: (1) they take as input a list (or in some cases lists) of peaks in a simple tab-delimited format, (2) perform a given analysis, and (3) output one or more peak lists. These modules can be used in any order, since their input and output are peak lists of the same format (i.e., *chromosome*, *start position*, *end position*). Thus, the novelty of the framework is the capability to combine these modules and design specific workflows that enable multi-step bioinformatics analyses of ChIP-seq data, according to the user's objectives and hypotheses. Figure 1 shows two scenarios that combine some of the ChIPseeqer modules. These scenarios are indicative examples based on our experience in analyzing several ChIP-seq datasets, and others could be considered as well. For example, a potential workflow (see Figure 1A) involves:

**Table 1:** The main tools of the ChIPseeqer framework.

Tool name	Description	GUI availability
QcAnalysisTools	Offers basic quality control tools.	NA
ChIPseeqerSplitReadFiles	Splits read files (e.g., bed, eland) into one read file per chromosome.	√
ChIPseeqer	Peak detection algorithm.	√
ChIPseeqerSummaryPromoters	Creates a promoters-based annotation of the detected peaks (i.e., gene name-description, peaks)	√
ChIPseeqerAnnotate	Finds the peaks distribution in the genome (e.g., exons/introns/intergenic) and creates lists of these peaks.	√
ChIPseeqerPeaksTrack	Creates a UCSC Genome Browser track for the detected peaks.	√
ChIPseeqerMakeReadDensityTrack	Creates a UCSC Genome Browser track for the reads density.	√
ChIPseeqerNongenicAnnotate	Finds the peaks that overlap with repeating elements, CpG islands and segmental duplicates.	√
ChIPseeqerFIRE	Runs FIRE for the detected peaks, in order to perform an unsupervised motif discovery.	√
ChIPseeqerMotifMatch	Runs MyScanACE for the detected peaks, in order to look for specific motifs (Jaspar, Bulyk PBM databases).	√
ChIPseeqeriPAGE	Runs PAGE for the genes associated with the detected peaks, in order to perform pathways analysis.	√
ChIPseeqerPathwayMatch	Looks for genes (and their corresponding peaks) that are associated to a specific pathway (e.g., apoptosis, GO:0060742).	√
ChIPseeqerCons	Estimates the conservation scores for the detected peaks and for random intervals to allow comparison.	√
ChIPseeqerDensityMatrix	Creates a reads density matrix for a window around the TSS or the TES of the genes, or for any interval selected.	NA
ChIPseeqerReadCountMatrix	Estimates the avg/max reads number for every input peak, across multiple ChIP-seq datasets and creates a peak-based reads matrix.	NA
ChIPseeqerCluster	Clusters a matrix (e.g., k-means, hierarchical, SOMs) and visualizes the clustering.	NA
CompareIntervals	Compares two lists of peaks and finds the overlapping peaks and the peaks that are unique in each list.	√
CompareGenes	Compares two lists of genes and finds the common genes and the genes that are unique in each list.	√
ChIPseeqerComputeJaccardIndex	Estimates the Jaccard similarity coefficient for a set of peak files. The larger the coefficient, the more similarity you have between two peak files	√
ChIPseeqerMakeGenepartsMatrix	Creates gene-based matrices (one for promoters, one for exons, etc) for many peak files. Summarizes the number of peaks that fall in specific gene parts, across many different peak files (TFs).	NA
ChIPseeqerFindDistalPeaks	Finds peaks that are away from known genes.	NA
ChIPseeqerFindClosestGenes	Finds the closest gene(s) for each peak.	NA
ChIPseeqerGetReadCountInPeakRegions	Estimates the avg/max reads number for every peak, for a ChIP-seq dataset and creates a peak-based read matrix.	NA
FindPeaksWithMotif	Extracts the peaks that have a specific FIRE motif (can be applied after running FIRE).	NA
MakePAGEInput	Creates the input file for iPAGE from a list of genes.	NA

The table shows the names of the tools, short description of their functionality and their availability within the ChIPseeqer interface. This is not an exhaustive list; all available tools are documented online [37].

(1) running the peak detection algorithm for a TF (e.g., ETS) ChIP-seq dataset,

(2) finding the peaks that have a specific motif (e.g., the ELK1 motif) using the *ChIPseeqerMotifMatch *module,

(3) identifying the peaks that bind at the promoters of known RefSeq genes using *ChIPseeqerAnnotate*, and

(4) performing pathways analysis on these genes with *ChIPseeqeriPAGE*, in order to find biological processes in which the given TF is likely involved. Another workflow (see Figure 1B) identifies putative enhancers based on TF and histone modifications ChIP-seq data. In that case, the intergenic peaks are first detected using *ChIPseeqerAnnotate*, and then the peaks that also overlap with enhancer marks [42] are reported using *CompareIntervals*. The corresponding subset of peaks represents putative enhancers; to discover informative regulatory elements within these peaks, unsupervised de novo motif analysis can be performed using *ChIPseeqerFIRE*. Finally, the *ChIPseeqerCons *module can be used to compare the conservation between putative enhancers and random genomic regions, in order to determine enhancers that are most likely to be functional.

### Use of ChIPseeqer - Example

To illustrate the power and flexibility of ChIPseeqer, we analyzed a published ETS1 ChIP-seq [43], performed in Jurkat T cells. ETS1 is an oncogene [44] and member of the ETS family of eukaryotic transcription factors. It is preferentially expressed at high levels in B and T cells, and plays a critical role in T cell activation [45]. Recent studies based on chromatin immunoprecipitation have shown ETS1 binding events in both promoters and enhancers in Jurkat T cells [43,46].

### ETS1 binds to thousands of locations and is associated with binding sites of other TFs

**Figure 2:**
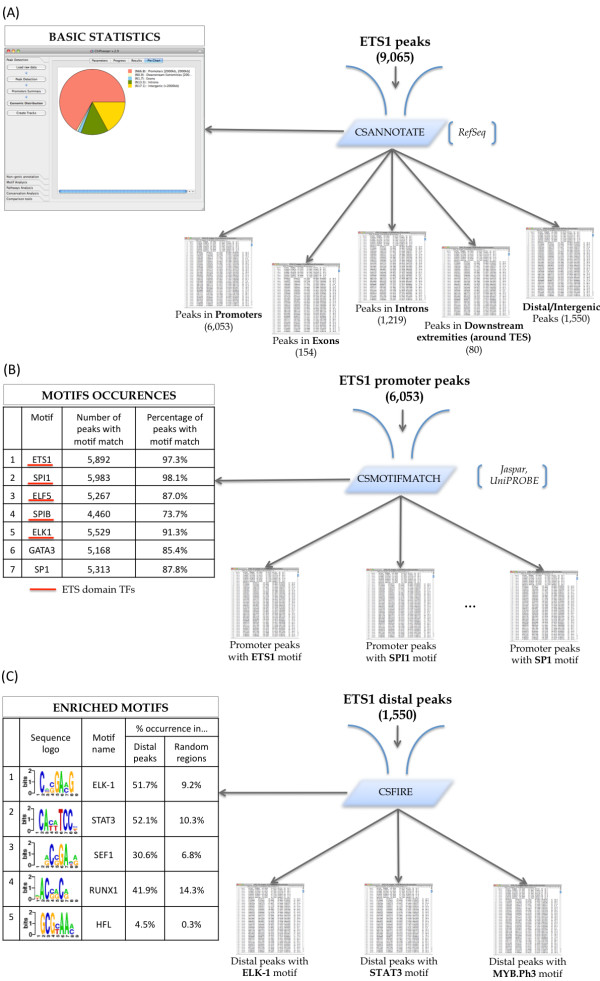
**Analysis of the ETS1 ChIP-seq dataset**. (A) The *ChIPseeqerAnnotate *module outputs the distribution of the ETS1 binding peaks in gene parts, as well as several lists of peaks that were found in a specific gene part (e.g., promoters, exons, introns). (B) The occurrence of specific motifs among the ETS1 peaks is shown, after using *ChIPseeqerMotifMatch*. The underlined motifs represent transcription factors of the ETS domain. (C) Unsupervised motif discovery, using *ChIPseeqerFIRE*, reveals multiple motifs that derive from the same regions. The fraction of ETS1 peaks containing at least one instance of each motif is given, with the expected frequency of the motif in the random regions given in the parentheses.

Our peak detection algorithm identified 9,065 ETS1 peaks. We associated these peaks with genes using the *ChIPseeqerAnnotate *module and the RefSeq annotation (Figure 2A). This analysis revealed a large occupancy of ETS1 peaks at the promoters of the genes (~67%), but also that ETS1 binding occurs on intergenic regions (~17%), at least 2 kb away from known TSS. Using the lists of these *promoter peaks *and *distal peaks *automatically generated by *ChIPseeqerAnnotate*, we performed "supervised" motif analysis (using known motifs) and *de novo *motif discovery. In the supervised analysis on the promoter peaks *ChIPseeqerMotifMatch *module), we determined subsets of ETS1 peaks that contain motif occurrences for other ETS family members (e.g., SPI1, SPIB, ELK1) [43,47,48], using motif weight matrices from the JASPAR [49] and UniPROBE [50] databases. *ChIPseeqerMotifMatch *reveals that a large fraction of ETS1 peaks (more than 73%) contain such matches (see Figure 2B). The unsupervised analysis (*ChIPseeqerFIRE *module) for the distal peaks revealed that multiple motifs appear from the same regions, among which ETS-domain motifs (e.g., ELK1), but also motifs resembling binding elements recognized by non-ETS related factors (see Figure 2C). For example, the HLF motif, which is bound by the hepatic leukemia factor and has been implicated in childhood B-lineage acute lymphoid leukemias, was also found in the intergenic ETS1 peaks. A motif resembling the AML-1a/RUNX1 binding sites was discovered as well using this *de novo *analysis. RUNX1 is a TF associated with several types of leukemia and is known to bind to T cell receptor enhancers [43]. The RUNX1 association with ETS1 distal peaks led us to look for ETS1 binding in putative enhancers as described in the next section.

### ETS1 binds to many putative enhancers

**Figure 3:**
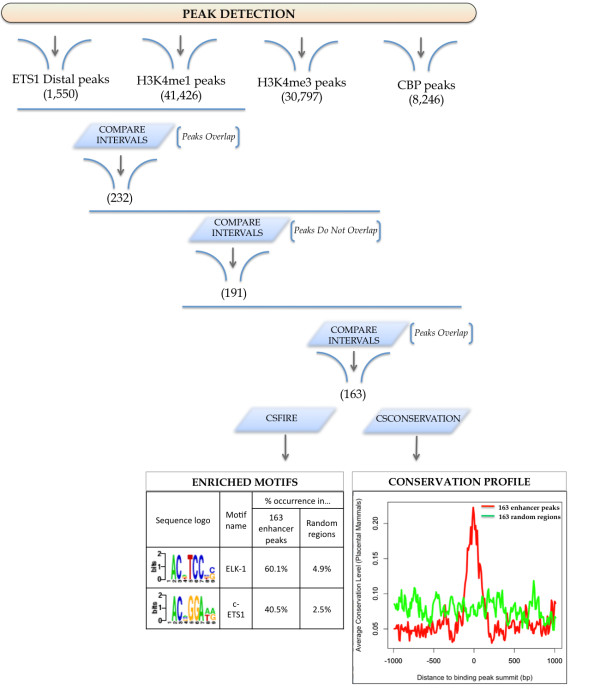
**Identification of putative enhancers**. This workflow shows the identification of putative enhancers, by progressively filtering the distal peaks with histone modification enhancer marks (i.e., presence of H3K4me1 and absence of H3K4me3) and CBP binding. De novo motif discovery and conservation analysis were then performed, which showed highly enriched ETS-domain motifs and high conservation scores in the set of putative enhancers compared to random regions.

To identify and characterize putative enhancers among the ETS1 peaks, we use the list of *distal peaks *obtained from the *ChIPseeqerAnnotate *analysis. To better identify enhancers, we also analyzed the CBP ChIP-seq dataset described by Hollenhorst et al. [43] (also Jurkat T cells), as well as the ChIP-seq histone marks datasets of primary CD4^+ ^T cells described by Barski et al. [51]. CBP protein shares regions of very high sequence similarity with p300, a protein that binds to many enhancers [52]. Moreover, several studies [42,53,54] have suggested high levels of H3K4me1 combined with low levels of H3K4me3 as a signature for predicting enhancers. Our peak detection algorithm identified 8,246, 41,426 and 30,797 enriched regions for CBP, H3K4me1 and H3K4me3 datasets respectively. In order to locate the putative enhancers, we looked for the ETS1 distal peaks that have histone signature for enhancers--presence of H3K4me1 and absence of H3K4me3-- and are also co-occupied by CBP. Using the ChIPseeqer *CompareIntervals *module, we identified such peaks (see Figure 3). First, we determined all ETS1 distal peaks (1,550) that also overlap with at least one peak in the H3K4me1 dataset (232 peaks). Second we looked for peaks that have absence of H3K4me3 marks (191 peaks), and finally, we determined which of the remaining peaks (i.e., ETS1 distal peaks with H3K4me1 but without H3K4me3 marks) overlap with at least one CBP peak (163). Statistical assessment of the overlaps described here showed that not all of them were different from chance expectation (data not shown). However, in what follows, the 163 peaks obtained from this analysis are considered to be putative ETS1-binding enhancers. We then performed unbiased motif discovery for this set of putative enhancers, using the *ChIPseeqerFIRE *module. This analysis revealed over-representation of two ETS domain-related motifs, the ELK-1 and the c-ETS motifs (see Figure 3) in the putative ETS1-binding enhancers peaks, and under-representation in the random regions. Finding these highly enriched motifs in such a small subset of peaks (that is ~0.18% of the initial pool of peaks and ~10% of the ETS1 distal peaks), but not in random regions, shows that the putative enhancers were not arbitrarily identified.

Moreover, to examine whether these regions are also conserved (and thus probably functional), we performed conservation analysis using the *ChIPseeqerCons *module. One of the capabilities of this module is to determine the conservation profile in and around the ChIP-seq peaks, using the phastCons scores [55]. The conservation profiles in randomly selected regions were also determined, in order to compare the level of conservation between the input peaks and randomly generated genomic regions. After averaging the conservation profiles in the two groups (i.e., peaks and random regions), higher level of conservation was indeed noticed for the group of putative enhancers, as can be seen in Figure 3. In this example, we showed how the identification of putative enhancers could be performed in ChIPseeqer, starting from the distal peaks and progressively filtering them with histone modification enhancer marks and CBP binding. De novo motif discovery and conservation analysis were then performed, which revealed highly enriched ETS-domain motifs and high conservation scores for the set of putative enhancers compared to random genomic regions. To further investigate the biology of ETS1 binding in Jurkat T cells, we also looked for potential biological pathway differences between the genes associated with ETS1 binding peaks in promoters (6,053 peaks) and with ETS1 binding peaks in intergenic regions (1,550 peaks). We used the *ChIPseeqeriPAGE *module, in order to find pathways that can discriminate the two groups of genes, (i.e., pathways enriched in one group but not in the other). We used the two lists of genes as input, one list for each group. Using the Gene Ontology annotation, we noticed a higher enrichment of T and B cell related pathways [43] in the distal peaks group, such as leukocyte differentiation, lymphocyte activation, immune response, immune system development and others (Table 2), rather than in the promoter peaks group. We also observed similar results using SignatureDB [56], a database of gene expression signatures mainly derived from B and T cells. In particular, we found a significantly higher enrichment of many T cell-related pathways and gene sets in the distal peaks compared to the promoter peaks (Table 2), such as the signatures "Tcell_PIind_CsAdown4x" [57] and "Thymic_SP_CD4+Tcell_gt_Blood_CD4+Tcell" [58].

**Table 2:** Pathways analysis between the ETS1 distal and promoter peaks.

		**Distal Peaks**	**Promoter peaks**
	
**T/B cell related**	**Gene Ontology**		
	Leukocyte differentiation, GO:0002521	**p < 0.001**	p < 1
	Lymphocyte activation, GO:0046649	**p < 0.001**	p < 1
	Hemopoiesis, GO:0030097	**p < 0.001**	p < 1
	Hemopoietic or lymphoid organ development, GO:0048534	**p < 0.001**	p < 1
	Immune response, GO:0006955	**p < 0.01**	p < 1
	Immune system development, GO:0002520	**p < 0.01**	p < 1
	B cell proliferation, GO:0042100	**p < 0.01**	p < 1
	B cell activation, GO:0042113	**p < 0.001**	p < 1
	
**Others**	Biopolymer catabolic process, GO:0043285	p < 1	**p < 1e-29**
	RNA splicing, GO:0008380	p < 1	**p < 1e-50**
	DNA metabolic process, GO:0006259	p < 1	**p < 1e-29**
	
**T/B cell related**	**SignatureDB**		
	Tcell_PIind_CalciumDefPtdown4x_Feske_Fig6	**p < 1e-05**	p < 1
	CD40_upregulated_Burkitt_lymphoma	**p < 0.001**	p < 1
	CD40_downregulated_Burkitt_lymphoma	**p < 0.01**	p < 1
	Pax5_repressed	**p < 0.01**	p < 1
	Tcell_PIind4x_Feske_Fig6	**p < 1e-08**	p < 1
	Tcell_PIind_CsAdown4x	**p < 1e-04**	p < 1
	
**Others**	Ribosomal_protein	p < 1	**p < 1e-06**
	Myeloma_PR_subgroup_up	p < 1	**p < 1e-05**

The table shows some of the pathways and lymphoma-related signatures that were found enriched in the distal peaks and the promoter peaks groups. The distal peaks group was highly associated with T cell and B cell related ontologies and signatures, while for the promoter peaks group more general and housekeeping categories were enriched. The Gene Ontology and the SignatureDB gene expression signatures were used for this analysis (*ChIPseeqeriPAGE *module). The p-values for each pathway for both groups are also shown.

The former signature originates from a study focusing on the signalling pathways network downstream of the T cell receptor [57], explaining the gene expression changes during T cell activation, whereas the latter signature comes from the analysis of phenotypic and functional parameters of T cell differentiation stages by studying human thymocytes, an important organ of T cell production [58]. On the other hand, the promoter peaks group was highly associated with more general pathways and gene sets, such as RNA processing, RNA splicing, metabolic process, proliferation and others (Table 2). These results are consistent with previous findings [43], where ETS1 bound intergenic regions were associated with genes involved in T cell specific functions, while ETS1 occupancy in promoters occurred at genes related to housekeeping functions. Using *CompareGenes*, a tool in our framework that allows comparisons on the gene level, we also looked for genes that have ETS1 peaks in their promoters (6,053 promoter peaks) *and *in intergenic regions (163 putative enhancer peaks). This analysis gave us 39 genes (see Table 3). One hypothesis that can be formed is that binding of ETS1 at both promoters and enhancers of these 39 genes mediates looping of the distal elements onto proximal promoters. Thus, these genes may be regulated by ETS1 through a chromatin looping event. This prediction can be further tested using chromosome conformation capture based techniques [59-61]. In summary, using the ChIPseeqer framework on a published ETS1 ChIP-seq dataset we showed that:

**Table 3:** List of the 39 genes with both promoter and distal ETS1 peaks.

# Gene ID Gene Description
AKAP11 A-kinase anchor protein 11 2
AKR1A1 alcohol dehydrogenase 3
ATP5O ATP synthase subunit O, mitochondrial precursor 4
C1orf109 hypothetical protein LOC54955 5
C2orf29 hypothetical protein LOC55571 6
C9orf123 transmembrane protein C9orf123 7
CDK9 cell division protein kinase 9 8
CHSY1 chondroitin sulfate synthase 1 9
CKAP2L cytoskeleton-associated protein 2-like 10
CLINT1 clathrin interactor 1 11
DUSP2 dual specificity protein phosphatase 2 12
DUSP6 dual specificity protein phosphatase 6 isoform 13
HSPC157 hypothetical LOC29092 14
KIAA0427 CBP80/20-dependent translation initiation factor 15
LDHA L-lactate dehydrogenase A chain isoform 5 16
LOC100188949 hypothetical LOC100188949 17
LOC285456 hypothetical LOC285456 18
LSM14B protein LSM14 homolog B 19
MAX protein max isoform a 20
MRPS18A 28S ribosomal protein S18a, mitochondrial 21
MTF2 metal-response element-binding transcription 22
NAIF1 nuclear apoptosis-inducing factor 1 23
NDUFA10 NADH dehydrogenase [ubiquinone] 1 alpha 24
POMP proteasome maturation protein 25
PSMA6 proteasome subunit alpha type-6 26
RBM16 putative RNA-binding protein 16 27
RBM38 RNA-binding protein 38 isoform a 28
RPN1 dolichyl-diphosphooligosaccharide--protein 29
SEPHS2 selenide, water dikinase 2 30
SIRPG signal-regulatory protein gamma isoform 1 31
SPRED2 sprouty-related, EVH1 domain-containing protein 32
TFRC transferrin receptor protein 1 33
TMEM18 transmembrane protein 18 34
TRIP13 thyroid receptor-interacting protein 13 isoform 35
TXN2 thioredoxin, mitochondrial precursor 36
UBE2D2 ubiquitin-conjugating enzyme E2 D2 isoform 1 37
ZFAT zinc finger protein ZFAT isoform 1 38
ZNF212 zinc finger protein 212 39
ZNF683 zinc finger protein 683

The table shows the 39 genes that were found to have both promoter and intergenic ETS1 peaks. It is possible that ETS1 binding at the promoters and enhancers of these genes is explained by looping of the distal elements onto proximal promoters. This hypothesis could be tested using chromosome conformation capture based techniques.

• There is a large occupancy of ETS1 peaks at the promoters but also at intergenic regions.

• ETS1 regions are bound by multiple motifs, either from the ETS-domain or non-ETSrelated (e.g., HLF, RUNX1).

• Specific pathways are preferentially related to genes with ETS1 binding in promoters or intergenic regions.

• It is straightforward to characterize ETS1 binding to genomic regions with enhancers signatures (e.g., H3K4me1+/H3K4me3-).

• It is possible to determine a list of genes that may be regulated by an enhancer-bound

TF, through a chromatin-looping event. Although these analyses could have been performed by combining several published tools or custom scripts, in ChIPseeqer this is fast (see *Performance Evaluation *section in Additional file 1) and straightforward and does not requiring any programming knowledge. Thus, by creating custom workflows that combine powerful computational programs, ChIPseeqer facilitates the comprehensive and in-depth analysis of ChIP-seq datasets.

### ChIPseeqer modules

This section demonstrates the diversity and versatility of the framework by presenting basic ChIPseeqer modules in further detail. A more exhaustive description of these tools is available online [37].

### ChIPseeqerAnnotate: Gene-level annotation of peaks

This module associates a set of ChIP-seq peaks, given as input, with the closest genes in the genome, and determines where each peak is located in these genes. In particular, *ChIPseeqerAnnotate *classifies input peaks in categories, (i.e., promoter, distal, intergenic, intronic, exonic and downstream). This module generates lists of peaks found in each of these classes of genomic regions. This analysis is controlled by default or user-defined parameters controlled by the user, such as the promoter window around the TSS and the annotation database. For example, promoters are defined by default as 4kb-long regions, around the TSS but not extended further than the downstream extremity of the genes. Moreover, *ChIPseeqerAnnotate *reports peaks overlapping with the *first intron*, since it has been reported that some first introns play a vital role in transcriptional control and splicing [62]. The tool also determines peaks not overlapping with any gene part, but found to be at least at a user-defined distance away from known genes (default is set to 2 kb away). We call these peaks distal or intergenic, because they occur in intergenic regions (known to contain important regulatory elements such as enhancers and insulators). The lists of peaks generated by *ChIPseeqerAnnotate *can be directly used in other tools within the framework to perform subsequent analyses. This can be useful to focus further analyses on specific classes of peaks (e.g. promoter peaks or intergenic peaks); *ChIPseeqerAnnotate *lets users extract these subsets of peaks. *ChIPseeqerAnnotate *has other interesting features. It generates a gene-based matrix that summarizes the number of peaks found in each gene part (e.g., promoters, exons, introns, intergenic) and can help the user identify quickly the peak binding occurring in their gene of interest. We have also developed tools that merge and combine this matrix output, in order to extract, for example, the genes with both promoter and intergenic peaks. The expected fraction of peaks in different genomic categories (e.g., promoters, introns, exons, intergenic) is also provided, based on the fraction of the genome in each of these regions, so that it can be compared to the observed fraction of the input peaks in each category. Finally, several widely used gene annotations are supported, such as the RefSeq, Ensembl, UCSCGenes, and AceView (other databases can also be easily added). This feature enables comparing the peak-gene association results between different databases.

### Pathways analysis modules

Pathways analysis can help elucidate important biological mechanisms associated with genome-wide binding and histone modification patterns. and can be performed after peaks have been associated with genes, as described in *ChIPseeqerAnnotate*. We have integrated two pathways analysis modes in the framework that involve: (1) looking for a given pathway of interest within the genes associated with input peaks and (2) looking for any pathways that are enriched in these genes. Pathways annotations are obtained from the Gene Ontology [63], KEGG database [64], Biocarta pathways [65], the SignatureDB online resource [56], and the Reactome pathways [66]. Importantly, both modes generate lists of peaks associated with genes in the query pathway or in the enriched ones. These peaks can then be used as input to other tools in the framework.

#### 1. ChIPseeqerPathwayMatch: User-specified pathway analysis

**Figure 4:**
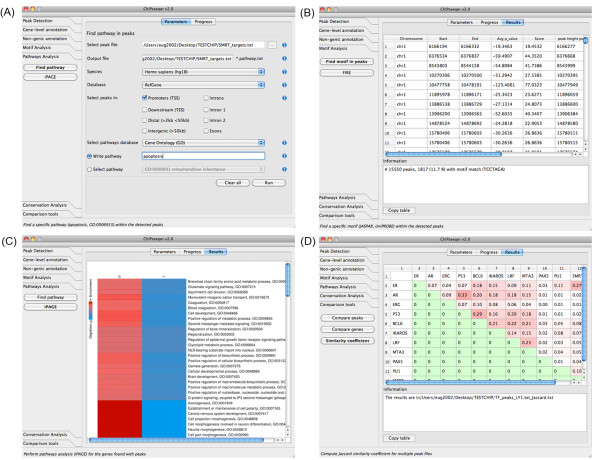
**ChIPseeqer graphical interface**. (A) The users can control all parameters of the tools. For example, in the *Find Pathway *tool (the GUI version of *ChIPseeqerPathwayMatch*) the user can select: the input peaks, the species of their data, the gene annotation database used to extract the genes related to the input peaks, which subset of the peaks to include in the analysis (e.g., promoter peaks, intergenic peaks), and which pathways database to use in order to look for the pathway. The desired pathway can be either selected from a list of available pathways or typed by the user (e.g., apoptosis, development). (B) The typical output of each tool is a table summarizing all peaks resulting from the analysis, as well as basic statistics (e.g., how many peaks found). Here, the peaks that contain the *TCCTAGA *motif are shown, after using the *Find Motif in peaks *tool (the GUI version of *ChIPseeqerMotifMatch*). (C) Several tools also provide graphical output. For example, the summary result of *iPAGE *tool (the GUI version of *ChIPseeqeriPAGE*) is a pathway enrichment table showing the level of enrichment for all pathways found in the genes related to the input peaks (category 1), compared to the genes used as background (category 0). (D) The output of the *Similarity coefficient *tool (the GUI version of *ChIPseeqerComputeJaccardIndex*) is a color-coded matrix, showing the pairs of datasets that have more common peaks than others, with darker red color.

When using this tool, the user first selects which of the input peaks to include in the analysis, based on their association with genes. For example, only peaks that belong to promoter regions can be included (Figure 4A). Alternatively, all peaks can be included, irrespective of where they reside within gene regions. Then, the user either selects the desired pathway from a list of available pathways in the provided pathway database (e.g Gene Ontology), or directly enters a pathway name (e.g., apoptosis, GO:0060742). *ChIPseeqerPathwayMatch *then finds all genes that belong to the selected pathway and outputs the corresponding ChIP-seq peaks. These peaks can then be used as input to other modules (e.g., for regulatory elements analysis). The hypergeometric distribution is used to assess the statistical significance of the pathway association: it determines whether the input peaks are associated with more genes in the query pathway than expected by chance (see Additional file 1).

#### 2. ChIPseeqeriPAGE: Pathways analysis using iPAGE

In order to discover highly enriched pathways in the genes associated with input peaks, we use iPAGE [67], an information-theoretic pathway analysis framework. In iPAGE, sets of genes are used as input, and pathways that are enriched in each set are reported [67]. *ChIPseeqeriPAGE*, is the module that integrates iPAGE within user-defined workflows. As with the previous module, users choose which input peaks to include in the analysis, based on their association with genes. The program then outputs the lists of peaks associated with specific enriched pathways.

### Regulatory element analysis modules

Regulatory element analysis of ChIP-seq peaks can discover the DNA sequence motifs bound by the TF assayed by ChIP-seq, and/or to find sequences bound by its co-factors. We have integrated two regulatory element analysis modes in the framework: (1) analysis based on known motifs or user-defined motif patterns, and (2) *de novo *motif analysis. Both analyses require extracting DNA sequences under the peaks from the genome reference sequence. Efficient extraction is performed by pre-indexing genomes using the SAMTools C library [68].

#### 1. ChIPseeqerMotifMatch: User-specified regulatory element analysis

Several software tools support searching for peaks that match a specific motif, but they often have limitations that restrict their usability. For example, in Cistrome [20] it is not straightforward to look for a specific motif, since available motifs are not shown to the user (only the available motif databases are). In HOMER [30], only motifs previously detected by the software are available for searching; the integration with public and popular sequence motif databases such as JASPAR that would enlarge the pool of available motifs is limited. *ChIPseeqerMotifMatch *seeks to overcome some of these limitations. To perform known motif analysis in *ChIPseeqerMotifMatch*, the user either selects the desired motif from a compiled dataset of ~250 TF binding sites (defined as position-specific weight matrices) from JASPAR [49] and UniPROBE [50] databases, or provides a consensus sequence in the form of regular expressions (e.g., TCCAAT, [AT]CG[CT]). In the former case, peak regions are scanned using the Berg and Von Hippel method [69] and a user-defined affinity threshold [70], and peaks containing one or more occurrence of the motif are given as output. Additional information such as motif positions within the peak and orientation are also reported. In the latter case, user-specific consensus sequences are used instead of weight matrices, and peak regions are scanned using regular expression matching algorithms from the pcre C library [71].

#### 2. ChIPseeqerFIRE: De novo regulatory element analysis

*De novo *motif analysis is performed using FIRE, an information-theoretic methodology for identification and characterization of regulatory elements [72]. In order to search for any informative motifs that are highly enriched within the detected ChIP-seq peaks, background sequences are first created. These background sequences can be extracted either randomly across the entire genome (option "random"), or immediately adjacent to the peak regions (option "adjacent"). They can also be extracted so as to preserve the C+G and CpG content of the input sequences using two different options. The "CGI" option estimates the fraction of the original peaks that overlap with CpG islands, and then produces random background regions that maintain this fraction of CpG islands overlap. Alternatively, using the "1MM" option, the program calculates for each input peak sequence 1st order Markov frequencies and uses these frequencies to generate new random sequences. As shown in Additional File 1 Figure S10, both CGI and 1MM preserve C+G and CpG frequencies of the input peaks. The option that the users should use depends on the question they want to address (e.g., Are there are any DNA motifs enriched in ChIP-seq peaks compared to regions flanking these peaks?, Are there any DNA motifs enriched in ChIP-seq peaks compared to randomly selected genomic regions with similar lengths and nucleotide compositions?). After identification of the motifs that best explain the distinction between peak regions and background sequences, peak lists containing each motif are extracted and can be used as input to other tools in the framework, such as pathway analysis tools.

### ChIPseeqerNongenicAnnotate: Nongenic peak annotation

The ability to integrate the results of a ChIP-seq study with existing and publicly available ChIP-seq is an important. For example, this integration could suggest transcription factor, co-factors or histone modification that should be further explored because of their extensive overlap with a set of ChIP-seq peaks. In the ChIPseeqer framework, the *ChIPseeqerNongenicAnnotate *provides such capabilities; it can determine the subset of input peaks that overlap with peaks obtained from TF or histone modification ChIP-seq datasets of the ENCODE project [73,74], as well as the statistical significance of this overlap (ENCODE datasets are subject to the ENCODE data usage policy available at http://genome.ucsc.edu/ENCODE/terms.html). *ChIPseeqerNongenicAnnotate *can perform additional integrative analyses. For example, extensive literature has shown that TF binding sites and specific histone modifications can be associated with repeated elements [75] and other nongenic elements, such as CpG islands [76]. Filtering the peaks based on that type of features could reveal interesting groups of peaks that have the potential to alter and impact gene expression (e.g., possible promoters, retroelements that impact transcriptional networks [75] and more). Using the *ChIPseeqerNongenicAnnotate *module and track-based data from the UCSC Genome Browser, users can quickly and easily determine which of their input ChIP-seq peaks overlap with: (1) known repeated sequences (identified by RepeatMasker [77]), (2) CpG islands and (3) segmental duplications. While such comparisons can be performed via the UCSC Table Browser (i.e., use *intersection *between any two tracks), *ChIPseeqerNongenicAnnotate *facilitates these analyses and allows their integration with other analyses within the framework.

### ChIPseeqerCons: Conservation analysis

Cross-species conservation analysis is necessary in order to discover functional genomic elements (e.g., distal regulatory elements) and also to prioritize the most promising genomic elements for experimental validation. For these reasons, we have developed *ChIPseeqerCons*, a tool that estimates the conservation for a given set of peaks and outputs the peaks whose average conservation score is greater than a user-defined threshold (default is set to 0.5). The most useful aspect of this module, is estimating the conservation level of sequences adjacent to the input peaks, or of randomly selected sequences, thus allowing global assessment of peak conservation.

Another interesting feature of *ChIPseeqerCons *is producing conservation profiles for regions around the summit of the peaks (default is 2kb-long regions), and for random intervals: the average conservation score is estimated for every *n*-sized bins of the regions (default is n = 10 nucleotides). By plotting the resulting conservation profiles, we can easily compare the level of conservation between the input peaks and randomly generated genomic regions (see Figure 3). *ChIPseeqerCons *uses the phastCons [55] or phyloP [78] scores (freely available as tracks from the UCSC Genome Browser website), calculated from placental mammalian genomes or primates.

### Analysis of read density profiles

The analysis of read density profiles, when combined with clustering methods, can help identify groups of genes with similar binding profiles in their promoters, or groups of peaks that tend to have similar histone modification or TF binding patterns. In ChIPseeqer, we have developed tools that take as input a set of genomic regions and: (1) calculate the read density profile of the regions (split regions into bins and calculate the average read count within each bin), (2) count the maximum or average number of ChIPseq reads for each genomic region. These tools can also perform RPKM-style read count normalization [79] prior to read counting, in order to compare multiple experiments with different numbers of short reads.

#### 1. ChIPseeqerDensityMatrix: Read density matrix

*ChIPseeqerDensityMatrix *lets the users explore and analyse the average read density profiles, either for user-defined regions around the TSS or TES of the genes (default is set to 4 kb around the TSS), or around the summit of the given peaks (default is set to 2 kb window centred to the peak summit). For each region, bins of *n *nucleotides (default is 10 nucleotides) are created and the average number of reads for each bin is counted. For example, if 4 kb regions are extracted around the TSS of genes, the result of this module will be a matrix that contains for each promoter the average number of reads for 400 bins of 10 nucleotides each. Clustering the promoters of the resulting matrix is then performed based on their read density profiles, using either the built-in Self-Organizing Map algorithm [80], or by interfacing with Cluster 3.0 software [81,82] (*ChIPseeqerCluster*). The results can be directly visualized using Postscript/PDF heatmaps produced using our built-in visualization tools or using TreeView [83] (included in the framework). Lists of genomic regions for each cluster can be exported and then used as input into other tools, in order to answer questions such as "Are there any regulatory elements associated with a given promoter binding pattern?" and "Which pathways discriminate between promoter binding patterns?".

#### 2. ChIPseeqerReadCountMatrix: Read count for multiple ChIP-seq experiments

*ChIPseeqerReadCountMatrix *estimates for each of the input peaks the maximum or average number of reads for multiple ChIP-seq datasets. The result of this module is a matrix that contains, for each of the input peaks, the maximum or average reads count for every ChIP-seq experiment. Similarly, the *ChIPseeqerCluster *module can be used to cluster the peaks of this matrix based on the reads number across multiple datasets, in order to reveal groups of peaks that share common binding in several TFs or histone modifications. The clusters of peaks can be extracted and used as input into other tools.

### Integration and comparison of ChIP-seq experiments

As more and more ChIP-seq datasets become publicly available, the need for data integration and comparison is becoming essential. Such integration can reveal how different TFs cooperate to regulate gene expression [84], as well as the interplay between TF binding and histone modifications [85,86]. The integration between ChIP-seq datasets can be realized by determining the overlap between sets of peaks. In ChIPseeqer, we have addressed this need by implementing fast interval tree-based tools for comparing ChIP-seq experiments at the peak level (*CompareIntervals*). These tools can be used to compare sets of peaks, and quickly: (1) identify overlapping peaks, (2) merge sets of peaks, or (3) determine peaks in the first set that do not overlap with any peaks from the second set (i.e. find unique peaks). Moreover, as described in previous section, these tools can assess the significance of the overlap between two sets of peaks using randomization tests that take into account the genomic distribution of peaks. In addition to simply counting how many peaks overlap between two peak lists, we provide tools that can also quantify the extent to which two sets of peaks overlap by estimating the pairwise Jaccard similarity coefficient between pairs of ChIP-seq datasets (*ChIPseeqerComputeJaccardIndex*). The Jaccard index is estimated as the number of peaks that overlap between two peak files, divided by the union of the two files. The larger the coefficient, the more similar two datasets are in terms of overlapping peaks (see Figure 4D). Such comparisons can also be performed at the gene level; we have developed similar tools for gene-based comparisons (*CompareGenes*) that can be easily used on the genes-based output of *ChIPseeqerAnnotate*. Finally, the annotation of peaks against a collection of ENCODE ChIP-seq datasets (*ChIPseeqerNongenicAnnotate*), as well as the read density analysis across multiple datasets (*ChIPseeqerReadCountMatrix*), both described in previous paragraphs, were also developed in the context of integration and comparison of multiple ChIP-seq experiments.

### Visualization tools

Visualization is tightly integrated to all modules of the framework in order to facilitate ChIP-seq data exploration and summarize the results of each analysis. ChIPseeqer includes tools for creating UCSC Genome Browser tracks representing peak location and genome-wide read densities. It also includes tools for drawing pie charts summarizing the genomic distribution of the peaks, creating motif and pathway enrichment tables and conservation plots (see Figure 1, Figure 4C). The output of clustering read density profiles can be visualized either using heatmaps, or 2D Kohonen maps [80]. Finally, we provide tools (*ChIPseeqerPlotAverageReadDensityInGenes*, *ChIPseeqerPlotAveragePeaksNumberInGenes*) for the visualization of reads density and peaks number in gene parts (e.g., promoters, exons, introns). The description of these tools and examples of the visualization they provide can be found at the ChIPseeqer tutorial [37].

## Discussion

The ChIPseeqer framework can considerably facilitate the bioinformatics analysis of ChIP-seq data by providing an integrated suite of computational tools that are fast, easy to use (no programming experience required), and can be combined with each other. The variety of tools and the flexibility offered by their parameters (Figure 4A) makes it possible to address most biological questions that are often raised when analyzing ChIPseq datasets. Notably, as demonstrated before, ChIPseeqer users can create personalized workflows in order to perform specific but sophisticated analysis, often requiring integration of multiple datasets (Figure 4D).

ChIPseeqer is a continuously developing project and we are actively working on implementing several additional components. For example, more species will be supported soon (e.g., *C. elegans, zebrafish, chicken, rat*), as well as more visualization components. Moreover, facilitating the integration of ChIP-seq data with gene expression data, obtained from microarray or RNA-sequencing (RNA-seq) experiments, also represents an important avenue for future improvement of the framework.

## Conclusions

In order to fill the gap between the identification of ChIP-seq peaks and the biological interpretation of the data, we have developed ChIPseeqer, a comprehensive computational framework that can be adapted to a user's needs and to the hypotheses of a ChIP-seq study. We showed that using the ChIPseeqer framework we can perform sophisticated analyses of ChIP-seq datasets (e.g., compare and integrate peak/gene lists), explore the data from multiple perspectives (e.g., conservation, motifs occurrence, pathways enrichment), and address specific biological questions, such as "*How do promoter peaks differ from distal peaks?*", "*Are there genes with both promoter and enhancer peaks?*". We believe that this framework will be of great assistance to investigators who wish to perform high-level analysis of genome-wide ChIP-seq datasets, but do not yet possess advanced computer programming skills.

## Availability and requirements

ChIPseeqer is freely available and can be downloaded at http://physiology.med.cornell.edu/faculty/elemento/lab/CS_files/ChIPseeqer-2.0.tar.gz The system requirements, instructions on how to install and run the software, and a detailed tutorial are also provided at http://physiology.med.cornell.edu/faculty/elemento/lab/chipseq.shtml The ETS1 and CBPdatasets used in this paper can be found at the GEO (GSE17954), and the H3K4me1 and H3K4me3 datasets are available at http://dir.nhlbi.nih.gov/papers/lmi/epigenomes/hgtcell.aspx 

## List of abbreviations used

kb: kilobase; ChIP-chip: Chromatin Immunoprecipitation followed by DNA microarray hybridization; ChIP-seq: Chromatin Immunoprecipitation followed by sequencing; GUI: Graphical user interface; RNA-seq: RNA sequencing; TES: Transcription end site; TSS: Transcription start site; TF: Transcription factor.

## Authors' contributions

EGG implemented the ChIPseeqer GUI version and parts of the command-line version, performed the analysis of the ChIP-seq datasets and drafted the manuscript. OE implemented parts of the command-line version, supervised the project and edited the manuscript. All authors read and approved the final manuscript.

## Supplementary Material

Additional file 1 **This file describes in detail the quality control analysis tools and the peak detection algorithm, implemented within the ChIPseeqer framework, as well as performance evaluation results for several tools of the framework**. Click here for file
