# Supplementary material for: An integrated ChIP-seq analysis platform with customizable workflows
Source: BMC Bioinformatics. 2011 Jul 7;12:277. doi: 10.1186/1471-2105-12-277 (PMC3145611; doi:10.1186/1471-2105-12-277)
Supplement: Additional file 1 — This file describes in detail the quality control analysis tools and the peak detection algorithm, implemented within the ChIPseeqer framework, as well as performance evaluation results for several tools of the framework. [file 1471-2105-12-277-S1.PDF]

# Additional file 1

# An integrated ChIP-seq analysis platform with customizable workflows

**Eugenia G Giannopoulou, Olivier Elemento**

### ChIPseeqerQC: Quality Control

Quality control analysis includes basic tools that can help to evaluate the quality of the ChIP-seq experiment. In particular, the number of unique and clonal reads (i.e., several identical reads that map to the exact same position in the genome) is reported; high number of clonal reads can indicate biased amplification of fragments due to small initial material or insufficient sonication and may suggest redoing the experiment. The nucleotide frequency of the fragment reads is also estimated, in order to help identify sequence bias produced by the protocol followed, or by the preferential sequencing of the instrument used towards GC-rich or AT-rich regions. Finally, the distribution of read coverage (i.e., average number of reads representing a given nucleotide in the reconstructed sequence) is computed genome-wide. An estimate about the presence or absence of high-coverage regions in a ChIP-seq dataset is desired because locally high coverage in sequencing suggests that the experiment has likely been successful.

### ChIPseeqer: Peak Detection Algorithm

In principle, the output of any ChIP-seq peak detection algorithm (e.g., MACS, PeakSeq, FindPeaks) can be used within the framework, as long as it follows the tab-delimited "*chromosome, start position, end position*" format. An important drawback of some algorithms lies in the assumption of stranded distributions of tags at peaks, causes low performance of these programs in detecting broad binding regions (e.g.,

some histone modifications) [1]. For this reason, we have developed a simple and fast peak detection algorithm that uses uniquely mapped reads (allowing up to 2 mismatches) and processes data on a chromosome-by-chromosome basis. The steps followed by the algorithm are shown in Figure S1. A read density map is constructed for the ChIP data (which consists of millions of short reads mapped to the genome), as well as for the control data if available, by extending the reads to the average length of the DNA fragments in the sequenced DNA library (e.g., 170 bp) and by counting the number of overlapping reads at each nucleotide position, also called observed reads count (Figure S1A). We also estimate the expected read count for each nucleotide position, by taking into account the total number of reads, the extended fragment length, the chromosome length as well as the chromosome mappability (so as to correct for the non-unique fraction of the genome, onto which no or fewer reads can be mapped) (Figure S1B). We have performed simulations by randomly shuffling read positions on the chromosomes and found that expected read counts are Poisson-distributed. Therefore, a Poisson distribution probability model is used to compare the observed with the expected read counts ($\lambda$ parameter) for both ChIP and control data (if available). Log-transformed Poisson p-values are calculated for each genomic position and used as normalized read counts (Figure S1C). Log-transformed p-values are also calculated independently for control data and subtracted from ChIP log values in order to obtain a normalized score (Figure S1D). The peak detection algorithm can work even when no control data is present, in which case the subtracted log p-values will be equal to 0. Contiguous stretches of >100bp above a given threshold *t,* which represents the significance negative log p-value and is set by default to 15 (i.e., 10^-15), are considered a peak.  Peaks closer than a given distance (*mindist* parameter, set to 100bp by default) are merged before the final output. In our experience, broad

domain detection (e.g., H3K36me3, H3K79me2) is best performed by tuning these parameters, in order to detect lower peaks and allow merging adjacent peaks into broader domains. The former is specified by the *–t* option (set to *15* by default and to *1-5* for domain detection), which allows including peaks that are not very "sharp", while the latter is specified by the *–mindist* option (set to *100bp* by default and to *1000-5000bp* for domain detection), which allows merging continuous enriched regions into a large peak. The impact of varying the *t* and *mindist* parameters on peak detection is illustrated in Figure S2 (unpublished B cell H3K36me3 ChIP-seq dataset). When control data is provided, it is further required that there is at least an m-fold enrichment (default 2-fold) of reads in the ChIP over the control data. Moreover, the peak detection method gives the option to remove clonal reads, so as to avoid including artifacts (i.e., when one read is artificially duplicated into several ones). Importantly, the algorithm reports the peak start and end coordinates, but also the peak summit location, defined as the highest read count in the peak region.

Finally, two empirical approaches are provided to measure the level of statistical significance achieved by estimating the False Discovery Rate (FDR). The first approach involves running the peak detection algorithm by switching between the ChIP and control samples in order to find control peaks over ChIP. The empirical FDR is then defined as the ratio of the number of control peaks to the number of ChIP peaks. The second approach can be used even when no control data is available, and involves randomly splitting the ChIP data in two sets, one of which is used as the ChIP data and the other as the pseudo-control data. The FDR in this case is defined as the ratio of the number of peaks detected in the pseudo-control analysis to the number of peaks detected in the real ChIP experiment. Because our peak detection algorithm

is simple and extremely efficient, FDR simulations can be performed in a few minutes and allows varying the score comparison threshold until a desired FDR is achieved.

**Performance Evaluation**

In this section we summarize the performance evaluation results for the ChIPseeqer framework. In particular, we discuss the comparison of some ChIPseeqer tools with similar programs, and we present the time needed to perform the analysis described in the section *Use of ChIPseeqer-Example*. Our testbed included one host, equipped with a 2.8GHz Intel Core 2 Duo CPU and 4GB of RAM, running Mac OS X (version 10.6.7). While performing each analysis, the host was idle, with no other user processes running.

**Peak Detection Validation**

ChIPseeqer has already been included in a performance evaluation of seven ChIP-seq analysis packages by Qin et al. [2]. The results showed a very high overlap of the peaks detected by ChIPseeqer with the peak lists produced by the other algorithms, which is a good first validation of our algorithm. Additionally, we compared the ChIPseeqer algorithm with MACS 1.4 [3] and CisGenome 2.0 [4], two established and widely used programs in ChIP-seq peak detection, in terms of the FDR estimation, the motif occurrences in the peaks, the position of the motifs within the peaks, as well as the running time. Because an exhaustive evaluation of the peak detection algorithm is beyond the scope of this study, we only present results from the analysis of the ETS1 dataset that is discussed in the manuscript. In the future, we plan to design and perform a comprehensive validation of a wide range of different ChIP-seq datasets, and a comparative evaluation including more peak detection algorithms.

***Quantity and quality of the detected peaks***

Figure S3A shows the number of detected peaks for the H3K4me3 (green), H3K4me1 (red), and ETS1 (blue) datasets, from MACS, CisGenome, and ChIPseeqer (with the

default threshold parameter *t=15*, and with *t=10*). MACS and CisGenome run with their default parameters. Note that the number of reported peaks alone is not an adequate indication of the quality of the peaks, since many false positives may be reported as well. The estimation of FDR (Figure S3B) shows the expected proportion of false positives among all significant observations. For example, in ETS1 dataset a large number of peaks does not necessarily indicate better performance. CisGenome and MACS, which detected more peaks than ChIPseeqer (*t=15*), had the highest FDR values. Importantly, in ChIPseeqer, we notice that: (1) when threshold *t=10* is used, the number of peaks is comparable to those reported from other methods, and (2) when the more conservative threshold (*t=15)* is used, ChIPseeqer reports fewer peaks but achieves lower FDR.

To sum up, we believe that a compromise between the threshold used and the number of detected peaks is needed, in order to ensure higher quality peaks. For this reason, after several tests with many different ChIP-seq datasets, we have set the default threshold to *t=15*. However, the users are free to change this parameter according to their needs.

***Peaks width and motif analysis***

The width of detected peaks is an important matter for motif searches, since large sequences away from the actual binding regions may add noise [2, 5]. Figure S4 displays the number of identified peaks for the ETS1 dataset, with *different* peak widths for each peak detection algorithm. Overall, MACS reported very large peaks (wider than the peaks reported from other methods), which are prone to noise during motif analysis. Although both ChIPseeqer and CisGenome have similar median peak widths (around 300bp), the average peak width in CisGenome is ~470bp, while in ChIPseeqer is 350bp, with lower standard deviation than CisGenome (~200bp instead

of ~360bp). We posit that ChIPseeqer reports peaks of smaller average size and standard deviation compared to the other methods, which is an indication of more accurate detection of the actual binding regions.

Motif enrichment can be a measure of the performance of ChIP-seq methods, since consensus motifs are usually enriched in the transcription factor binding sites. In order to evaluate the three algorithms, we compare the proportion of the peaks that contain the motif of interest. For longer sequences the probability of including false motif occurrences by chance increases, and as it is clear in Figure S4 the different peak detection methods produce peaks of various lengths. To adjust for this bias, we extracted same-width regions around the peak summit (i.e., 250bp), for all peak lists coming from CisGenome, MACS, and ChIPseeqer (run with $t=10$ and $t=15$). We then looked for the ETS1 motif from the JASPAR database [6] within these peak lists, using the *ChIPseeqerMotifMatch* module. ChIPseeqer achieved the highest percentage of peaks with ETS1 motif (Figure S5A).

Additionally, we calculated the position of the best motif match within the peaks (e.g., motif position 50% means that the best motif match was found in the middle of the peak). The box plot in Figure S5B shows that ChIPseeqer performs equally well with the other two algorithms, in terms of the positional accuracy of the exact binding position estimate.

Overall, the ChIPseeqer peak detection algorithm achieves a comparable, and in some cases better, performance to other peak detection algorithms, based on the estimation of the FDR and the motif analysis. More importantly, our algorithm has the advantage of fast running time (Figure S6), which is essential now that very large datasets are being produced. Specifically, for the ETS1 ChIP-seq dataset ChIPseeqer was 2.2 times faster than CisGenome, and 4.7 times faster than MACS.

**Runtime analysis**
We measured the time needed to perform the analysis described in the manuscript, in order to show that using ChIPseeqer it is not only straightforward, but also fast. Table S1 shows the running time of peak detection (in minutes) for the ChIP-seq datasets described in the example (i.e., ETS1, CBP, H3K4me1 and H3K4me3 datasets), as well as the size of each dataset. The ChIPseeqer peak detection step can be completed in less than two minutes for these datasets.

The modules *ChIPseeqerAnnotate*, *ChIPseeqerMotifMatch*, and *CompareIntervals*[1] required less than one minute to run (Table S2).

However, the *ChIPseeqerFIRE* and *ChIPseeqeriPAGE* modules, which are computationally intensive because of the iterative statistical nature of their core components, FIRE [7] and iPAGE [8], required approximately 20 minutes to run (Table S2). In particular, the FIRE and iPAGE algorithms include several time-consuming steps, such as the calculation of mutual information values, the evaluation of the information significance via randomization tests, the motif seeds optimization, as well as other statistical and conditional information tests (see Supplementary Material of [7, 8]).

Finally, *ChIPseeqerCons* also needed ~20 minutes to run for the specific dataset, because we exploited its capability to read directly from the compressed (zipped) conservation score files. The reason we offer this feature is because the conservation score files are very large in size (the compressed hg18 phastCons files are ~2.7Gb for placental mammals, and ~3.6 for primates), and will take even more space if they are decompressed. In our experience, it takes more than twice more time for ChIPseeqer to access compressed zipped files compared to unzipped ones, but the former requires

---

[1] The time reported for *CompareIntervals* was without the estimation of z-score.

much less disk space.

### *ChIPseeqerFIRE runtime analysis*

In this section, we provide an estimation of the running time needed for *ChIPseeqerFIRE,* one of the most time-consuming modules of the framework. We randomly extracted 1K, 10K, 20K and 30K peaks out of a ~35K peaks file (ETS1 ChIP-seq dataset discussed in the paper, run with different parameters in peak detection), in order to create peak sets of different sizes, and used these datasets to measure the time needed to run.

*ChIPseeqerFIRE* is a two-steps module. The first step involves extracting the genomic sequences under the input ChIP-seq peaks, from the genome reference (denoted with blue color in Figure S7). Efficient extraction is performed on pre-indexed genome sequences using the samtools C library [9], and therefore takes negligible time compared to the total running time of a typical motif analysis (Figure S7). The second step involves running the FIRE program (denoted with red color in Figure S7), which dominates the total processing time for two reasons. The first one is because FIRE performs an exhaustive exploration of motif space in order to find all independent sequence motifs that are either over-represented or under-represented within the input sequences compared to control sequences. The FIRE runtime is highly dependent on the biological complexity of the ChIP-seq peaks, and in particular on the number of DNA-binding factors that bind to the peak regions. The second reason is because FIRE spends a significant amount of processing time optimizing and assessing the statistical significance of each detected motif. Moreover, FIRE performs a variety of analyses to characterize the discovered motifs, including motif co-occurrence and co-localization analyses, motif identification by comparison to included motif database.

We have measured the time needed for both steps, for the four different size peaks sets (Figure S7). In general, the total running time of *ChIPseeqerFIRE* is linearly correlated to the input size. Notably, the *ChIPseeqerFIRE* runtime does not exceed 1h30min even when analyzing 30,000 ChIP-seq peaks.

### *ChIPseeqerCluster runtime analysis and statistical significance*

Table S4 shows the time and memory requirements for the hierarchical and k-means algorithms of Cluster3 [10], using different input data. The input datasets were produced by the *ChIPseeqerDensityMatrix* module, and correspond to read density matrices of 1K, 5K, and 20K genes (rows) and 400 average read density values (columns). In hierarchical clustering, time and memory increase exponentially when larger input data is used, while in k-means there is linear correlation of time and memory with the input (Table S4).

As far as the SOMs implementation is concerned (also available through the *ChIPseeqerCluster* module), Figure S8 shows the time and memory requirements of the algorithm for different sizes of input, as well as when changing the parameters. As expected, run time depends linearly on the size of the input data (Figure S8A). Moreover, running the algorithm with the same input data (10K) but with different x and y dimensions (Figure S8B), and iterations (Figure S8C), we also observe that time is linearly correlated to these parameters of the algorithm.

To measure the memory requirements of the program, we used Valgrind and found that the required memory is also linearly correlated to the input size (Figure S8D). However, in multiple runs of the algorithm using the same input (10K) but different number of iterations, the same amount of memory is always reported (i.e., 154.1MB). Similarly, we observed that the memory requirements are independent of the x and y dimensions used for the algorithm.

In the SOMs implementation we estimate the clustering "quality" as follows: (1) we first compute the Euclidean distance or Pearson correlation distance between each point (gene) and the center of the cluster to which the gene is assigned to, (2) we estimate the average of these distances *w(k)* within each cluster k (*w(k)* shows how "tight" a cluster tends to be: the lower the better), and (3) finally, we sum up all the *w(k)* values and divide by the number of clusters. Importantly, the algorithm generates several clustering partitions obtained from different random cluster initialization (i.e., random cluster assignments), and reports the clustering partition with smallest "tightness" value. On a complementary note, the biological relevance of clustering partitions obtained using *ChIPseeqerCluster* can be assessed using multi-group pathway analysis as implemented in iPAGE [8].

**Qualitative evaluation**

In order to evaluate the credibility of two other ChIPseeqer modules, *ChIPseeqerCons* and *CompareIntervals,* we compared their output with the results produced by the online Cistrome framework [11], which is widely used by the scientific community due to its integrative nature and the plethora of analysis tools that offers.

We applied *ChIPseeqerCons* to the set of 163 putative enhancers that we analyzed in the example discussed in the manuscript (*Figure 3*), in order to study their cross-species conservation using the hg18 PhastCons scores for placental mammals. *ChIPseeqerCons* automatically calculated average, minimum and maximum conservation scores for each of these peaks (optionally peaks with average, maximum or minimum conservation below a given threshold can be filtered out). In addition, the tool produced average conservation profile plots in 2kb windows centered on peak summit. By default, an identical number of randomly located genomic regions were also generated, and their average conservation profile was added to the plot shown in Figure S9A. By including randomly located regions as control background,

*ChIPseeqerCons* lets users directly determine whether their input peaks are likely to be functional. A similar analysis with Cistrome produced the same average conservation plot for the 163 enhancers (Figure S9B); however we had to manually enter 163 randomly located regions and generate another conservation plot for these regions (Figure S9B). Moreover, Cistrome does not calculate the average conservation score of each region, making peak filtering based on conservation and further analysis of the most highly conserved peaks impractical.

We then compared our *CompareIntervals* tool to the Intersect tool in Cistrome/Galaxy [11], which allows finding overlaps between genomic intervals. Using *CompareIntervals*, the overlap between the ETS1 distal peaks (1,550) and the H3K4me1 peaks (41,426) described in the manuscript (also shown in *Figure 3*) consisted of 232 peaks *(z=63.48, p<1e-4).* Using *CompareIntervals* with the ANDNOT option, we found that 191 of these peaks did not overlap with the H3K4me3 peaks (30,797). We repeated the exact same analysis using the same peaks and the Intersect and Subtract Cistrome/Galaxy tools and found exactly the same number of peaks (232 and 191, respectively). These two independent analyses demonstrate the soundness of our interval tree-based comparison tool.

**ChIPseeqerPathwayMatch statistical significance**

In ChIPseeqerPathwayMatch module, we use the hypergeometric distribution to estimate the statistical significance of the pathway association:

$$P(X \geq k) = \sum_{i=0}^{k} \frac{\binom{m}{i}\binom{N-m}{n-i}}{\binom{N}{n}}$$

In the equation above, *k* equals the number of genes associated with the ChIP-seq

input peaks, and also associated with the given pathway, *m* is the number of genes assigned to the pathway, *n* is the number of all genes associated with the ChIP-seq input peaks, and *N* is the total number of genes.

**ChIPseeqeriPAGE statistical significance**

In iPAGE, pathway significance is calculated using an information-theoretic approach followed by randomization tests to estimate significance [8]. Briefly, the genes associated with a given set of ChIP-seq peaks are first determined using the *ChIPseeqerAnnotate* tool. A gene profile is then constructed, describing whether each gene in the selected gene annotation database (e.g., RefSeq) is associated to a peak or not. For a given pathway (e.g., cell cycle), a similar profile is constructed, describing whether each gene is known to be associated with the pathway or not. The mutual information (MI) between the two profiles is then calculated. The pathway profile is shuffled 10,000 times with the gene profile unchanged, and 10,000 shuffled information values are calculated. The number of times the shuffled MI is greater than or equal to the real one is calculated, thus providing a p-value. For a given p-value threshold, the false discovery rate is further calculated using additional randomizations. Further details are provided in the iPAGE publication [8]. Additionally, to quantify the level of over- and under-representation of an informative pathway in certain expression profiles, the hypergeometric distribution is used [8].

## References


1. Hawkins RD, Hon GC, Lee LK, Ngo Q, Lister R, Pelizzola M, Edsall LE, Kuan S, Luu Y, Klugman S, et al: **Distinct epigenomic landscapes of pluripotent and lineage-committed human cells.** *Cell Stem Cell* 2010, **6:**479-491.
2. Qin ZS, Yu J, Shen J, Maher CA, Hu M, Kalyana-Sundaram S, Chinnaiyan AM: **HPeak: an HMM-based algorithm for defining read-enriched regions in ChIP-Seq data.** *BMC Bioinformatics* 2010, **11:**369.
3. Zhang Y, Liu T, Meyer CA, Eeckhoute J, Johnson DS, Bernstein BE, Nussbaum C, Myers RM, Brown M, Li W, Liu XS: **Model-based analysis of ChIP-Seq (MACS).** *Genome Biol* 2008, **9:**R137.
4. Ji H, Jiang H, Ma W, Johnson DS, Myers RM, Wong WH: **An integrated software system for analyzing ChIP-chip and ChIP-seq data.** *Nat Biotechnol* 2008, **26:**1293-1300.
5. Wilbanks EG, Facciotti MT: **Evaluation of algorithm performance in ChIP-seq peak detection.** *PLoS One* 2010, **5:**e11471.
6. Sandelin A, Alkema W, Engstrom P, Wasserman WW, Lenhard B: **JASPAR: an open-access database for eukaryotic transcription factor binding profiles.** *Nucleic Acids Res* 2004, **32:**D91-94.
7. Elemento O, Slonim N, Tavazoie S: **A universal framework for regulatory element discovery across all genomes and data types.** *Mol Cell* 2007, **28:**337-350.
8. Goodarzi H, Elemento O, Tavazoie S: **Revealing global regulatory perturbations across human cancers.** *Mol Cell* 2009, **36:**900-911.
9. **Samtools** [http://samtools.sourceforge.net/]
10. de Hoon MJ, Imoto S, Nolan J, Miyano S: **Open source clustering software.** *Bioinformatics* 2004, **20:**1453-1454.
11. **Cistrome** [http://cistrome.dfci.harvard.edu/]

# Figures

### Figure S1 - Overview of the ChIPseeqer peak detection algorithm

(A) The number of the observed reads per nucleotide position is first counted. (B) The expected read count per position is then estimated taking into account the total number of reads, the fragment length, the chromosome length and mappability. (C) The Poisson model is used to compare the observed and expected read counts and compute a normalized score for each position based on p-values. (D) A peak is called when the normalized score is greater than a given threshold.

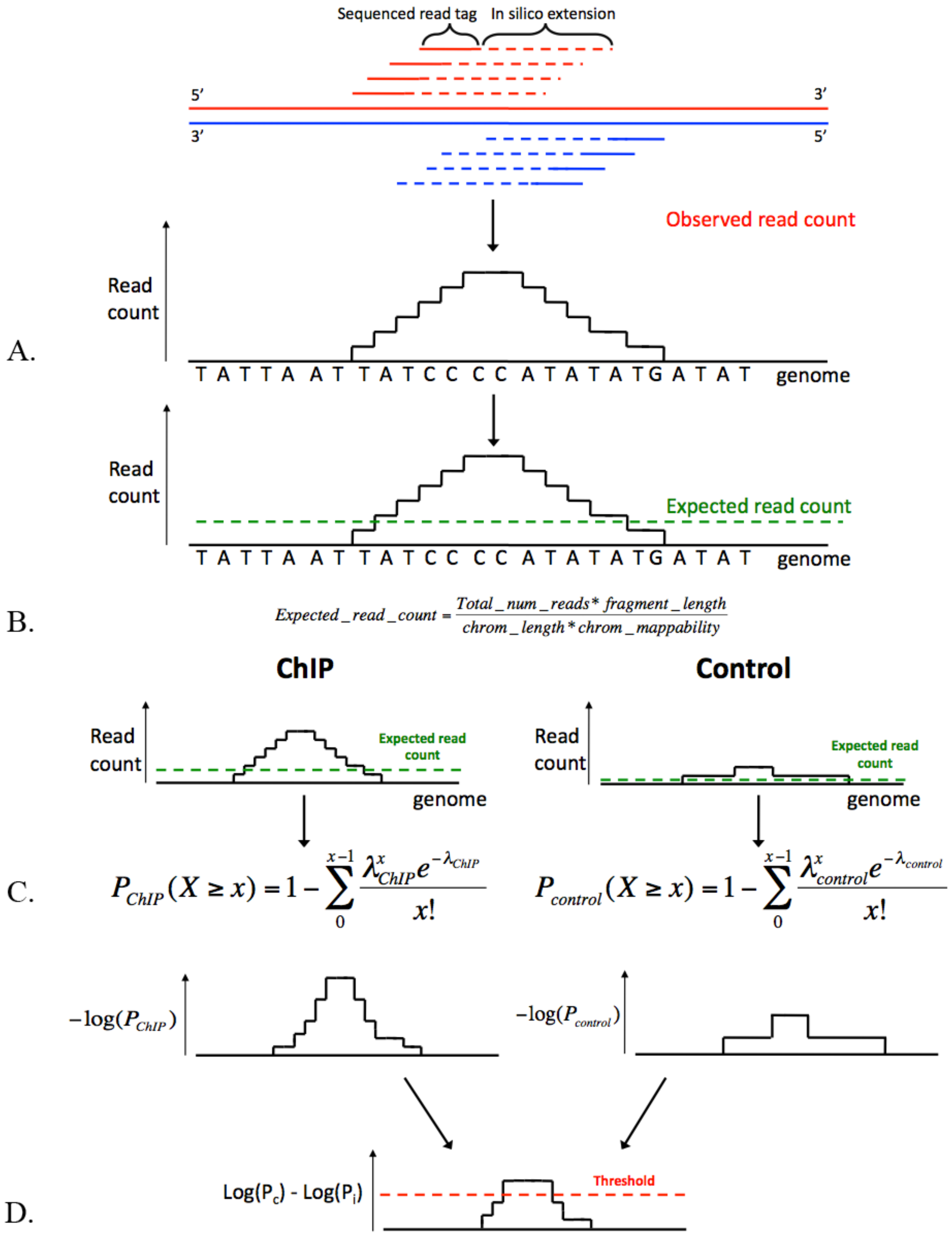
Sequenced read tag
In silico extension
5'
3'
3'
5'
Observed read count
Read count
A.
T A T T A A T T A T C C C C A T A T A T G A T A T
genome
Read count
Expected read count
T A T T A A T T A T C C C C A T A T A T G A T A T
genome
B.
$Expected\_read\_count = \frac{Total\_num\_reads * fragment\_length}{chrom\_length * chrom\_mappability}$
ChIP
Control
Read count
Expected read count
genome
Read count
Expected read count
genome
C.
$P_{ChIP}(X \geq x) = 1 - \sum_{0}^{x-1} \frac{\lambda_{ChIP}^{x} e^{-\lambda_{ChIP}}}{x!}$
$P_{control}(X \geq x) = 1 - \sum_{0}^{x-1} \frac{\lambda_{control}^{x} e^{-\lambda_{control}}}{x!}$
$-\log(P_{ChIP})$
$-\log(P_{control})$
D.
$\mathrm{Log}(P_c) - \mathrm{Log}(P_i)$
Threshold

**Figure S2 - Tuning the parameters of the ChIPseeqer peak detection algorithm, to capture broad domain peaks**

(A) By lowering the threshold *t,* which represents the significance negative log p-value and is set by default to 15 (i.e., 10^-15), we allow regions with lower reads density to be considered as peaks. The top band corresponds to threshold *t=1*, the middle one in *t=5*, and the bottom one in *t=10.* In higher *t* values, only "sharper" peaks are considered. (B) By increasing the minimum distance between the peaks, continuous enriched regions are merged into large peaks. The top band corresponds to *100bp* minimum distance, the middle one in *1000bp,* and the bottom one in *5000bp*.

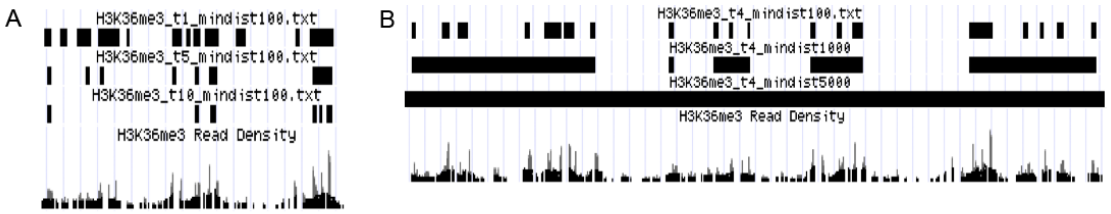

**Figure S3 - Quantity of peaks and false discovery rate of three different peak detection algorithms**

(A) Number of detected peaks shown for the H3K4me3 (green), H3K4me1 (red) and ETS1 (blue) datasets that are discussed in the paper. The methods tested were MACS, CisGenome and ChIPseeqer. ChIPseeqer was used with the default threshold parameter, *t=15* (def), and also with *t=10* (10). (B) The estimation of FDR involves switching between the ChIP and control samples, in order to find control peaks over ChIP. The empirical FDR is defined as the ratio of the number of control peaks to the number of ChIP peaks. Methods that report a large number of peaks do not necessarily perform better, since many false positives (high FDR values) may be included.

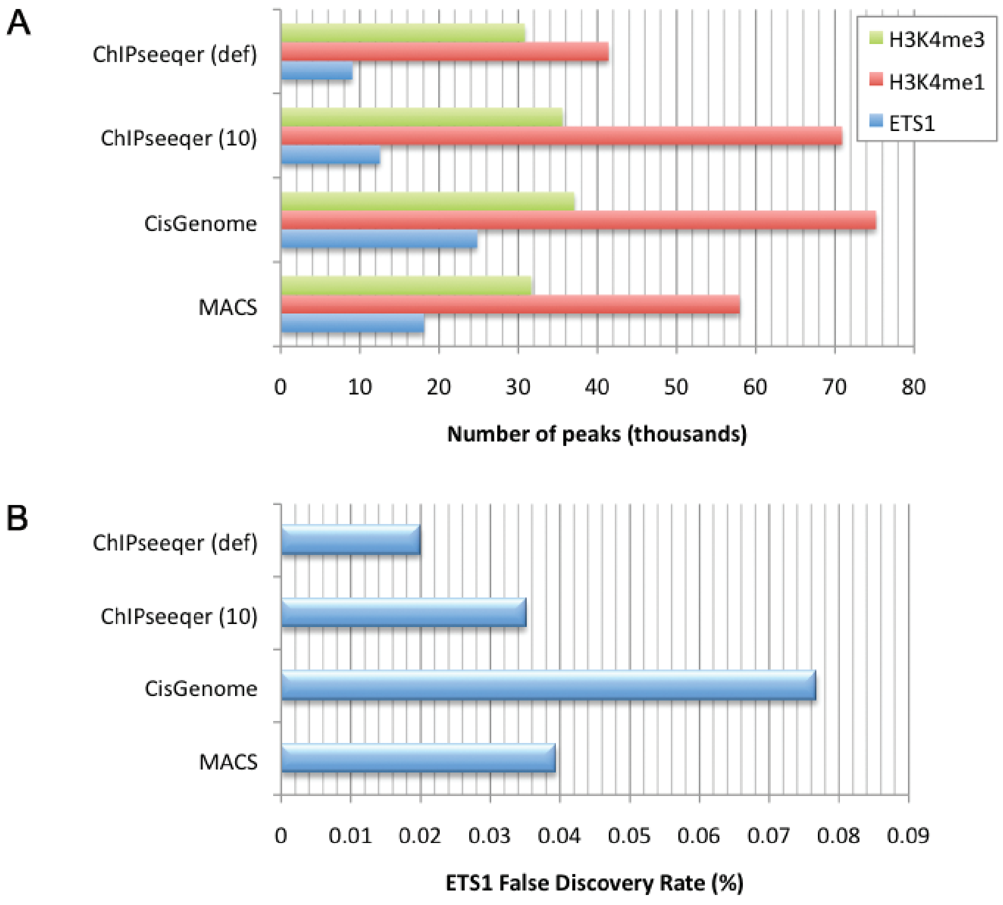

**Figure S4 - Width of ETS1 peak regions**

The plot displays the number of identified ETS1 peaks with different peak widths for each peak detection algorithm. The peak width is estimated as the difference between start and stop coordinates. The table shows the average and median peak width, and the standard deviation for all methods. Overall, MACS reported very large peaks compared to the other methods. ChIPseeqer and CisGenome have similar median peak widths, but ChIPseeqer reports peaks of smaller average size and standard deviation, which is an indication of more accurate detection of the actual binding regions.

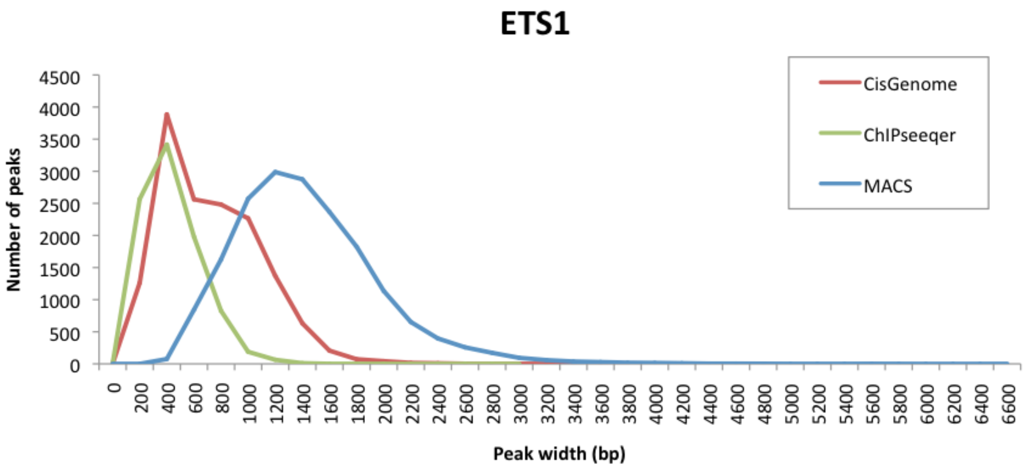


| | ChIPseeqer | CisGenome | MACS |
|---|---|---|---|
| **Avg. peak width** | 350 | 474 | 1333 |
| **Med. peak width** | 302 | 300 | 1265 |
| **Std. Dev.** | 203 | 359 | 535 |

**Figure S5 - ETS1 motif occurrences and positional accuracy**

(A) The graph displays the number of peaks with presence of ETS1 motif. Because longer sequences are more likely to contain the motif of interest by chance, we extracted regions of the same width (250 bp) around the peak summit for the peak lists of all methods. ChIPseeqer achieved the highest percentage. (B) The box plot shows the variation of the best motif match position within the peak. Motif position 50% means that the best motif match was found in the middle of the peak. In terms of positional accuracy, ChIPseeqer performs equally well with the other methods.

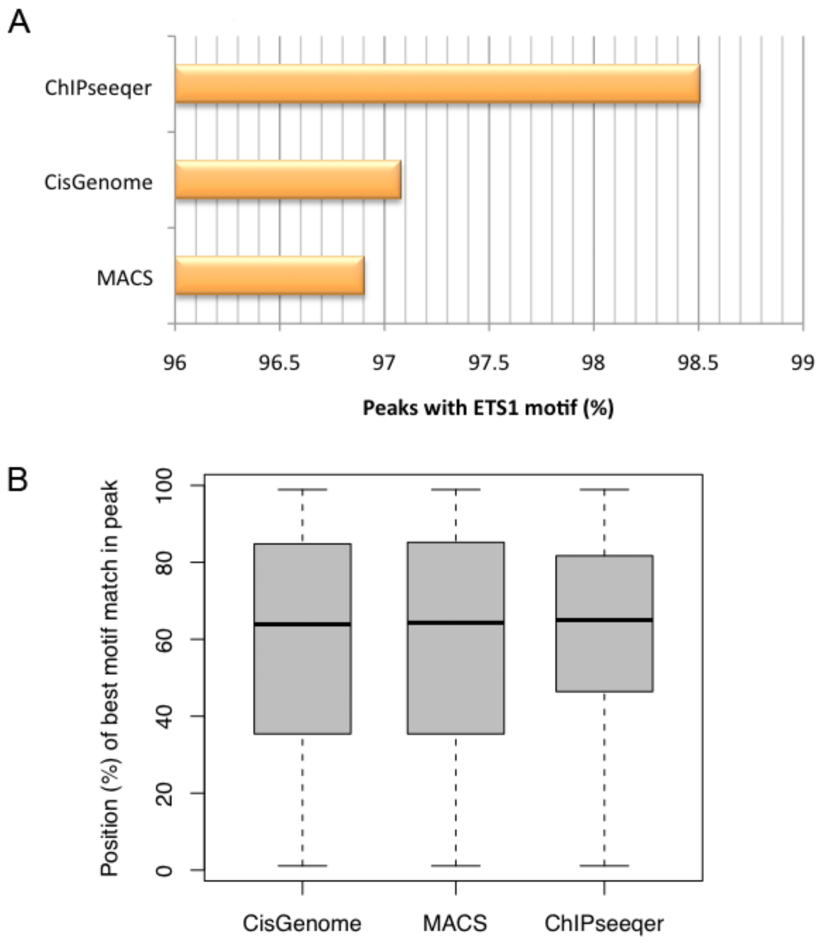

**Figure S6 - Run times comparison**

The graph shows the running time of MACS, CisGenome and ChIPseeqer peak detection algorithms for the ETS1 dataset. ChIPseeqer is 2.2 times faster than CisGenome, and 4.7 times faster than MACS.

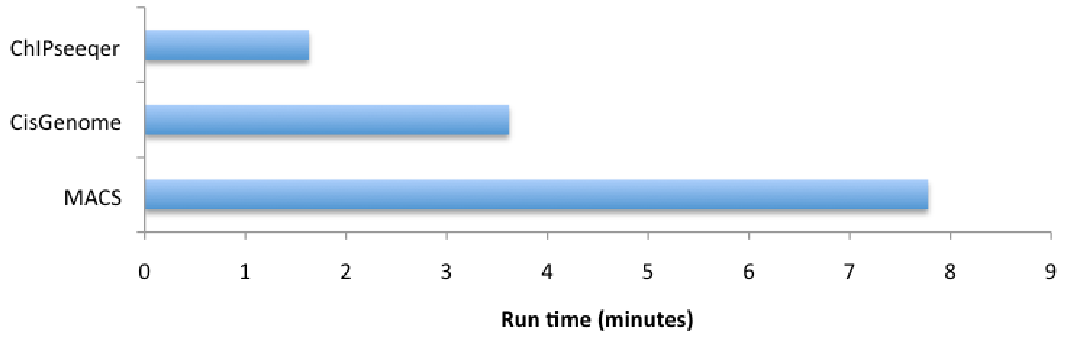

**Figure S7 - Run times for ChIPseeqerFIRE module**

The plot shows an estimation of run time for ChIPseeqerFIRE. The module includes the steps of: (1) sequences extraction (blue) and (2) run of the FIRE program (red). In general, the total running time of ChIPseeqerFIRE is linearly correlated to the input size. It is notable that the ChIPseeqerFIRE runtime does not exceed 1h30min even when analyzing 30,000 ChIP-seq peaks.

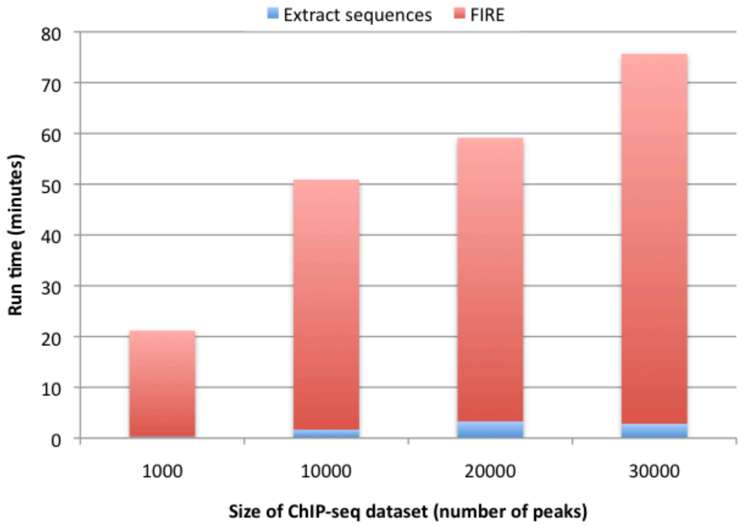

**Figure S8 - Time and memory requirements for ChIPseeqerCluster - SOMs**

The running time of SOMs algorithm is linearly correlated to: (A) the input size (run with parameters: 500 iterations, 3x4 dimensions), (B) the x and y dimensions of the SOM grid (run with input: 10,000 rows, and parameters: 500 iterations), and (C) the number of iterations (run with input: 10,000 rows, and parameters: 3x4 dimensions). (D) The memory requirements also depend linearly on the input size.

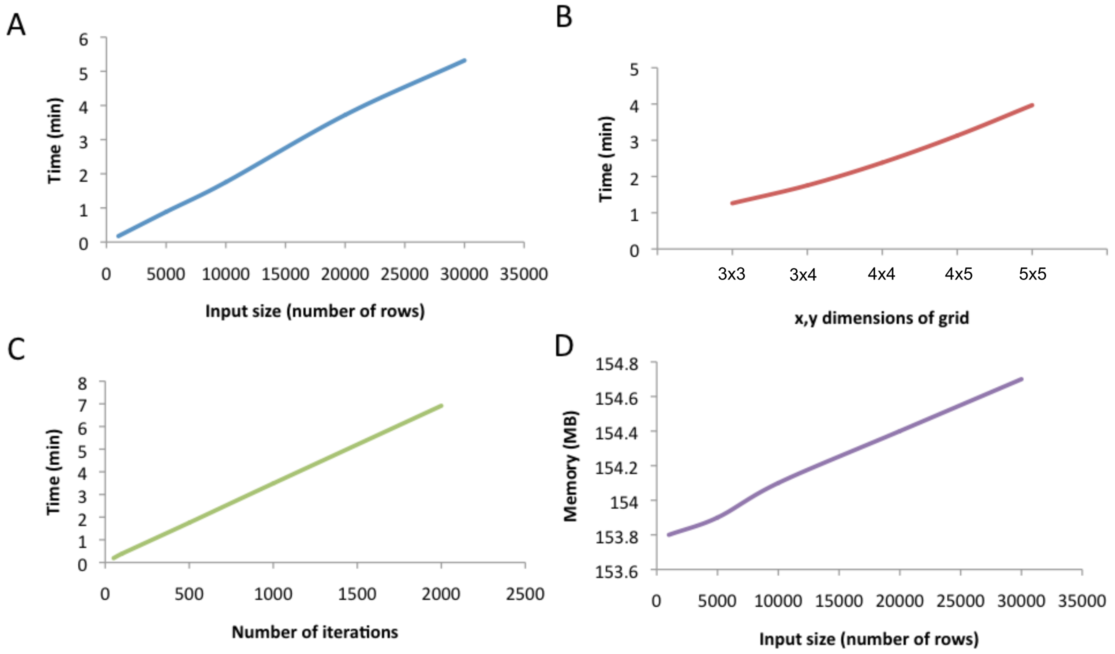

**Figure S9 - Conservation plots using the Cistrome toolbox and ChIPseeqerCons**

(A) The conservation plot created by ChIPseeqerCons for the set of 163 putative enhancers (red), and a set of 163 random genomic regions (green), which are discussed in the example (this plot is part of Figure 3 of the manuscript). (B) The conservation plots for the same set of enhancers (left), and random genomic regions (right), which are discussed in the example, created by the Conservation Plot tool of Cistrome. The conservation plots produced by the two tools are similar by visual examination: the conservation profiles have the same values range and similar rises and falls.

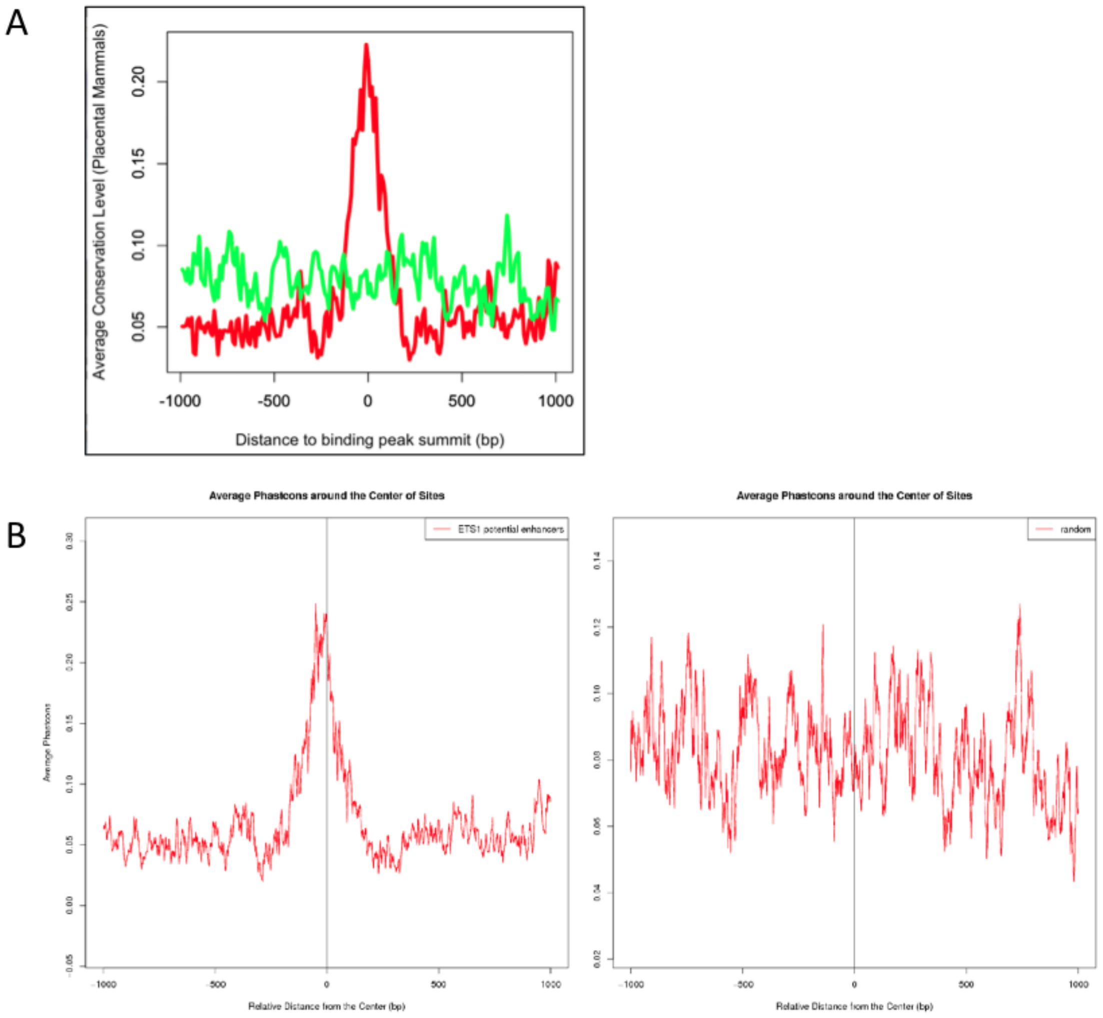

**Figure S10 - Number of C+G and CpG in original and randomly generated peaks**

The boxplots show (A) the number of G+C nucleotides, and (B) the number of CG dinucleotides, in the sequences of the original peaks, the random peaks using the “1MM” option, and the random peaks using the “CGI” option. We observe that there are no significant differences between the three populations, which suggests that both options produce background sequences that preserve the C+G and CG content of the original sequences.

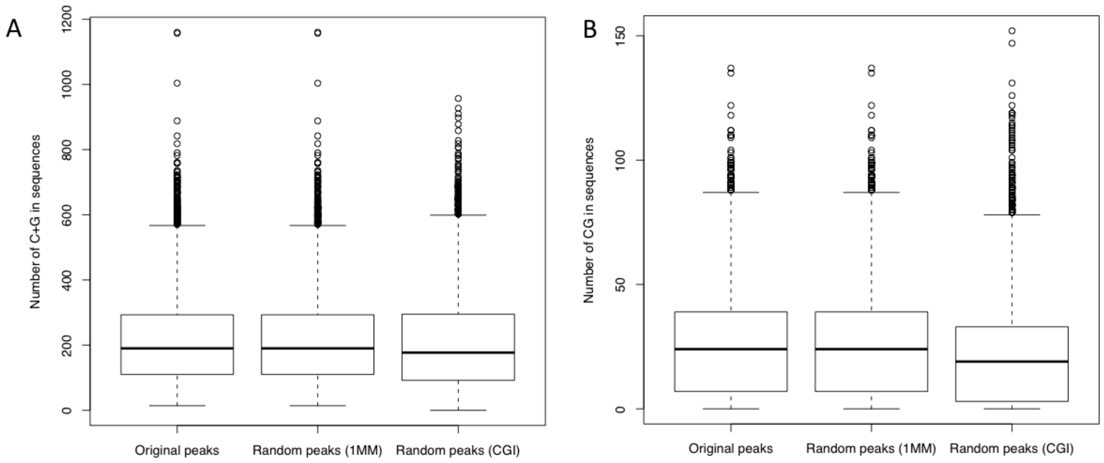

# Tables

### Table S1 - Peak detection runtime

The table shows the running time (in minutes) of peak detection for the ChIP-seq datasets described in the example. The size of the datasets in millions of reads is also shown. For the ETS1 and CBP datasets control ChIP-seq data (input) were also used.

| Module | Dataset | Size (reads) | Time (min) |
|---|---|---|---|
| ChIPseeqer.bin (peak detection) | ETS1 | ~7M (chip)\~15M (input) | 1.62 |
| | H3K4me1 | ~10M | 1.01 |
| | H3K4me3 | ~13M | 1.24 |
| | CBP | ~8M (chip)\~15M (input) | 1.77 |

### Table S2 - Runtime for the modules used in the example

The table shows the running time (in minutes) for the modules used to perform the analysis described in the paper, the size of the input data (number of peaks), and the corresponding figures that summarize the results in the manuscript. *ChIPseeqerAnnotate*, *ChIPseeqerMotifMatch* and *CompareIntervals* run in less than one minute. *ChIPseeqerFIRE*, *ChIPseeqeriPAGE* and *ChIPseeqerCons* need ~20 min to run; the former two because of their iterative statistical nature, and the latter because of its capability to read directly from compressed files. For the modules annotated with a star (*) two peak sets were used as input.

| Module | Details | Size (peaks) | Time (min) |
|---|---|---|---|
| ChIPseeqerAnnotate | ETS1 (Fig. 2A) | 9,056 | 0.656 |
| ChIPseeqerMotifMatch (ETS1) | ETS1 promoter peaks (Fig. 2B) | 6,053 | 0.02 |
| ChIPseeqerMotifMatch (SPI1) | ETS1 promoter peaks (Fig. 2B) | 6,053 | 0.02 |
| ChIPseeqerMotifMatch (ELF5) | ETS1 promoter peaks (Fig. 2B) | 6,053 | 0.025 |
| ChIPseeqerMotifMatch (SPIB) | ETS1 promoter peaks (Fig. 2B) | 6,053 | 0.045 |
| ChIPseeqerMotifMatch (ELK1) | ETS1 promoter peaks (Fig. 2B) | 6,053 | 0.023 |
| ChIPseeqerMotifMatch (GATA3) | ETS1 promoter peaks (Fig. 2B) | 6,053 | 0.021 |
| ChIPseeqerMotifMatch (SP1) | ETS1 promoter peaks (Fig. 2B) | 6,053 | 0.025 |
| ChIPseeqerFIRE | ETS1 distal peaks (Fig. 2C) | 1,550 | 22.05 |
| ChIPseeqerFIRE | ETS1 enhancers (Fig. 3) | 163 | 2.37 |
| ChIPseeqerConservation | ETS1 enhancers (Fig. 3) | 163 | 21.54 |
| CompareIntervals (AND)* | ETS1 distal/H3K4me1 (Fig.3) | 1,550/41,426 | 0.32 |
| CompareIntervals (ANDNOT)* | ETS1 dist_me1$^+$/H3K4me3 (Fig.3) | 232/30,797 | 0.12 |
| CompareIntervals (AND)* | ETS1 dist_me1$^+$_me3$^-$/CBP (Fig.3) | 191/163 | 0.03 |
| ChIPseeqeriPAGE (GO)* | ETS1 promoter/distal peaks (Table 2) | 6,053/1,550 | 19.36 |
| ChIPseeqeriPAGE (Staudt)* | ETS1 promoter/distal peaks (Table 2) | 6,053/1,550 | 5.453 |

**Table S3 - ETS1 ChIP-seq peaks with specific Jaspar motifs, using SeqPos and ChIPseeqerMotifMatch**

The table shows the number and percentage of the ETS1 ChIP-seq peaks reported to contain a specific motif from the Jaspar database. The *ChIPseeqerMotifMatch* module and the SeqPos tool from Cistrome were used with the same input parameters. In all cases, *ChIPseeqerMotifMatch* reported the same or a larger number of peaks than the *SeqPos* tool. In particular, *ChIPseeqerMotifMatch* found 28.8% (ELK1), 11.9% (SPIB), 21.9% (SP1), and 21.5% (SPI1) *more* peaks than SeqPos.

| **Jaspar motif** | **SeqPos (Cistrome tool)** | | **ChIPseeqerMotifMatch** | |
|---|---|---|---|---|
| ETS1 | 5,892 | 97.3% | 5,892 | 97.3% |
| ELK1 | 3,786 | 62.5% | 5,529 | 91.3% |
| SPIB | 3,745 | 61.8% | 4,460 | 73.7% |
| SP1 | 3,994 | 65.9% | 5,313 | 87.8% |
| SPI1/PU.1 | 4,684 | 77.3% | 5,983 | 98.8% |

**Table S4 - Time and memory requirements for Cluster3 Hierarchical clustering and k-means algorithms**

The time and memory requirements are shown for the hierarchical and k-means algorithms of Cluster3, using different input data: read density matrices of 1K, 5K, and 20K genes (rows) and 400 average read density values (columns). In hierarchical clustering, time and memory increase exponentially when larger input data is used, while in k-means there is linear correlation of time and memory with the input.

| **Hierarchical clustering (Pearson correlation/Pairwise linkage)** | **Time (minutes)** | **Memory (MB)** |
|---|---|---|
| 1000x400 | 0.0215 | 8.522 |
| 5000x400 | 1.146 | 118.8 |
| 20000x400 | 48.16 | 1617.92 |
| **k-means clustering (Pearson correlation/10 iterations/k=5)** | **Time (minutes)** | **Memory (MB)** |
| 1000x400 | 0.02 | 4.716 |
| 5000x400 | 0.248 | 23.4 |
| 20000x400 | 1.7 | 93.50 |
